# Whole Exome Sequencing Identifies Novel De Novo Variants Interacting with Six Gene Networks in Autism Spectrum Disorder

**DOI:** 10.3390/genes12010001

**Published:** 2020-12-22

**Authors:** Namshin Kim, Kyoung Hyoun Kim, Won-Jun Lim, Jiwoong Kim, Soon Ae Kim, Hee Jeong Yoo

**Affiliations:** 1 Genome Editing Research Center, Korea Research Institute of Bioscience and Biotechnology (KRIBB), Daejeon 34141, Korea; n@rna.kr (N.K.); kekgoo@gmail.com (K.H.K.); cerutx@gmail.com (W.-J.L.); 2 Department of Bioinformatics, KRIBB School of Bioscience, Korea University of Science and Technology (UST), Daejeon 34141, Korea; 3 Quantitative Biomedical Research Center, Department of Clinical Sciences, University of Texas Southwestern Medical Center, Dallas, TX 75390, USA; jiwoongbio@gmail.com; 4 Department of Pharmacology, School of Medicine, Eulji University, Daejeon 34824, Korea; 5 Department of Psychiatry, College of Medicine, Seoul National University, Seoul 03080, Korea; 6 Department of Psychiatry, Seoul National University Bundang Hospital, Gyeonggi 13620, Korea

**Keywords:** whole exome sequencing, autism spectrum disorder, de novo variants, korean cohort

## Abstract

Autism spectrum disorder (ASD) is a highly heritable condition caused by a combination of environmental and genetic factors such as de novo and inherited variants, as well as rare or common variants among hundreds of related genes. Previous genome-wide association studies have identified susceptibility genes; however, most ASD-associated genes remain undiscovered. This study aimed to examine rare de novo variants to identify genetic risk factors of ASD using whole exome sequencing (WES), functional characterization, and genetic network analyses of identified variants using Korean familial dataset. We recruited children with ASD and their biological parents. The clinical best estimate diagnosis of ASD was made according to the Diagnostic and Statistical Manual of Mental Disorders (DSM-5^TM^), using comprehensive diagnostic instruments. The final analyses included a total of 151 individuals from 51 families. Variants were identified and filtered using the GATK Best Practices for bioinformatics analysis, followed by genome alignments and annotation to the reference genome assembly GRCh37 (liftover to GRCh38), and further annotated using dbSNP 154 build databases. To evaluate allele frequencies of de novo variants, we used the dbSNP, gnomAD exome v2.1.1, and genome v3.0. We used Ingenuity Pathway Analysis (IPA, Qiagen) software to construct networks using all identified de novo variants with known autism-related genes to find probable relationships. We identified 36 de novo variants with potential relations to ASD; 27 missense, two silent, one nonsense, one splice region, one splice site, one 5′ UTR, and one intronic SNV and two frameshift deletions. We identified six networks with functional relationships. Among the interactions between de novo variants, the IPA assay found that the NF-κB signaling pathway and its interacting genes were commonly observed at two networks. The relatively small cohort size may affect the results of novel ASD genes with de novo variants described in our findings. We did not conduct functional experiments in this study. Because of the diversity and heterogeneity of ASD, the primary purpose of this study was to investigate probable causative relationships between novel de novo variants and known autism genes. Additionally, we based functional relationships with known genes on network analysis rather than on statistical analysis. We identified new variants that may underlie genetic factors contributing to ASD in Korean families using WES and genetic network analyses. We observed novel de novo variants that might be functionally linked to ASD, of which the variants interact with six genetic networks.

## 1. Introduction

Autism spectrum disorder (ASD) is a neurodevelopmental disorder characterized by abnormalities in social interaction and communication, with the presence of restricted interests and repetitive behaviors [1]. The estimated heritability of ASD is 64–91% [2]. Previous genetic etiology studies have analyzed families who have siblings with ASD, who are associated with a 25-fold higher risk of developing ASD than the general population [3]. Identifying the risk genes of siblings in patients with ASD is important. The development of ASD is influenced by both common and rare genetic variants. However, owing to extreme genetic heterogeneity, as suggested by the fact that a single gene aberration does not account for more than 1% of cases, identifying causal genes is challenging [4,5]. Furthermore, patients with ASD often present with other psychiatric, neurological, or physical co-occurring disorders, which suggest more complex genetic etiologies [6].

The first strategy to find risk factors of ASD involved identifying a significant number of common genetic variations in a large population. Recent genome-wide association studies (GWAS) [7,8,9] have identified several potential susceptible genes; however, the extreme innate heterogeneity of ASD limits the ability to identify most potential variants in such association approaches. Syndromic ASD is typically associated with chromosomal abnormalities or mutations in a single gene, and is known to be strongly associated with more than 100 genetic disorders (or factors), such as Rett and Fragile X syndromes [10]. However, genetic susceptibility to ASD varies among individuals, and may be derived from causative de novo rare variants. Recent studies on ASD have shown that rare variants contribute to identifying potential genes that increase an individual’s risk for developing ASD. Likewise, studies using next generation sequencing (NGS) have revealed several genes or variants associated with ASD.

In terms of de novo rare variants, evaluating individuals with ASD based on familial trio studies with relatively small sample sizes using NGS has been an excellent method to uncover the unknown genetic basis of ASD etiology. Mutations in hundreds of known genes, give rise to rare variants, that significantly increase the risk of ASD, and they are estimated to account for approximately 10–30% of autism cases [11]. Among these rare variants, some genes such as *NLGN1* [12], *VPS13B* [13], *GABRB3* [14], and *PTCHD1* [15], were found to increase ASD risk. Previous ASD research discovered that rare variants were found to be a novel de novo variant [16]. These variants have been reported to work in conjunction with common multi-gene risk variants [17] or to be associated with neuronal development [18]. There is little consideration of racial differences in recent studies using NGS [19]. While a panel sequencing study with 4503 target genes in Asians (Koreans) investigated associations with comorbidities, they did not target the de novo mutation nor analyzed the functional relationship of the identified genes [20]. Therefore, difficulties persist in identifying rare variants among patients of specific races due to the lack of consideration of the functional characteristics of the discovered variants in ASD.

This study aimed to examine rare de novo variants to identify the genetic risk factors of ASD using whole exome sequencing (WES). We investigated the functional characterization, genetic network analyses, and pathway profiling to understand the mechanism by which the involved genetic variants interact with each other and contribute to ASD based on that Korean familial dataset.

## 2. Methods

### 2.1. Subject Ascertainment and Ethnic Information

We recruited subjects with ASD and their biological parents. The best clinical diagnoses of ASD were made for probands and their siblings by board-certified child psychiatrists according to the Diagnostic and Statistical Manual of Mental Disorders (DSM-5^TM^) [21]. Comprehensive diagnostic assessments, including the Korean versions of the Autism Diagnostic Interview—Revised [22] and the Autism Diagnostic Observation Schedule [23] were used as diagnostic instruments. We used the Korean Educational Development Institute—Wechsler Intelligence Scale for Children—Revised (KEDI—WISC—R) [24] to examine overall intelligence and administered the Korean-Leiter International Performance Scale—Revised (K-Leiter—R) [25] to evaluate the probands’ nonverbal intelligence. Social ability was assessed using the Social Communication Questionnaire (SCQ) [26] and the Social Responsiveness Scale 2nd Edition (SRS-2) [27]. Detailed family history, in addition to environmental and social data, was collected from the parents of each proband. We excluded participants who had known chromosomal anomalies or declared having non-Korean biological parents from the study.

### 2.2. Study Design and Bioinformatics Analysis

We attempted to identify de novo mutations in patients with ASD at two separate stages. In the first exploratory stage (2013, high sequencing price), we performed two pooled sequencing from fathers’ and mothers’ DNA. The identified de novo variants were isolated and validated using Sanger sequencing. In the second stage (2016), we performed trio exome sequencing to identify de novo mutations (Supplementary Table S1).

**Figure 1:**
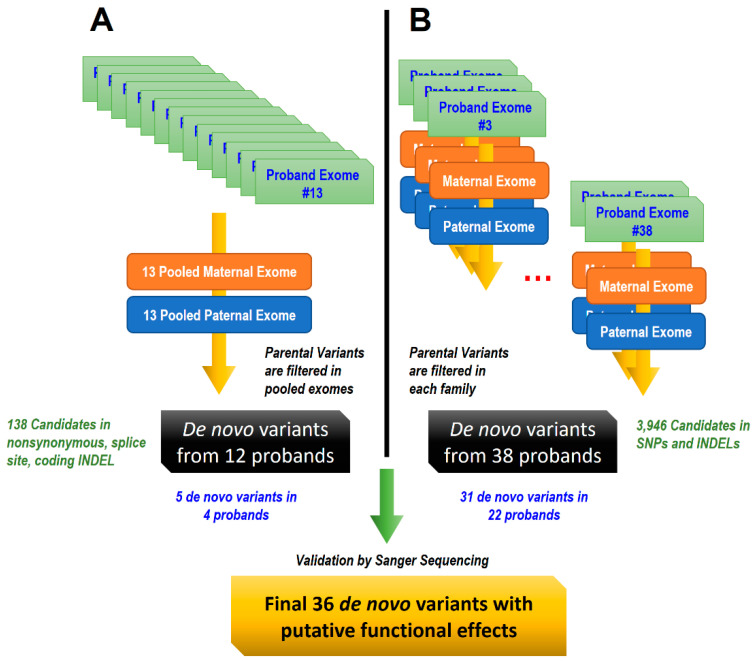
Design of a 51 Korean family trio cohort study. (**A**) First strategy: Variants from pooled parental exomes were removed to identify de novo variants from 13 probands. (**B**) Second strategy: Variants from both parental exomes were removed for each family.

First stage: In the first stage of this project, we collected the exome data of 13 probands and the pooled exome data of 13 fathers and 13 mothers (Figure 1A). In the pooled exome data, we expected at least 10X coverage for each parent. Selecting only de novo mutations of the 13 probands becomes feasible when all variants from the pooled exome data are removed. The average number of reads for the 13 probands was 107 million, and additional exome data were generated for the A5 and A9 probands. DNA samples from fathers and mothers were pooled considering sex chromosomes, which results in 472 and 501 million reads, respectively. DNA from parents of the A10 probands were not included in the pooled exome and we excluded the proband for further analysis. We found 138 de novo mutations (only for nonsynonymous, splice site, and coding INDELs) among 12 families. GATK Best Practices^TM^ was used for bioinformatics analysis. We carried out genome alignments and annotation to the reference genome assembly GRCh37 (hg19) using dbSNP 137 build databases. In silico prediction software such as SIFT, Mutation Assessor, and phyloP databases were also downloaded using the UCSC Genome Browser. All reads were mapped onto the reference genome using BWA v0.6.1. A series of analysis steps were performed on the BAM files, such as AddOrReplaceReadGroups, MarkDuplicate, and FixMateInformation modules in picard v1.79, additional BQSR, and IndelRealigner modules in the GATK v2.1 software. We used GATK UnifiedGenotyper to identify SNPs and INDELs. Lastly, variants with following values were filtered; “MQ >= 4 & MQ/DP > 0.1”, “QUAL < 100”, “QD < 5”, “FS > 60”, “MQ < 40”, “DP < 10”, and “GQ < 13”. After identifying of all de novo mutations by removing variants from the two pooled exome data, we selected SNPs using nonsynonymous, splice site, and coding INDELs. We prioritized all missense mutations if at least one of the following conditions were fulfilled: phyloP > 1.2, deleterious, as determined by the SIFT prediction, and high and medium functional impact, as determined by MutationAssessor.

Second stage: In the second stage, we collected exome data for the trio of 38 families, including male and female parents (Figure 1B). Two families (six exome datasets) were removed, since the family trio relationship was not met owing to mixed samples. An average of 76 million reads were produced, varying from 59 to 121 million reads. We identified de novo mutations by removing all variants from both parents. A total of 3946 de novo mutations (all SNPs and INDELs) were found in 38 families. We used the reference genome assembly GRCh37 (hg19) and dbSNP 138 to build databases. We also used GATK Best Practices in this step; however, the software versions were changed accordingly. We used BWA v0.7.12, Picard v1.119, and GATK v3.6. We performed annotations using the database available in ANNOVAR website (annovar.openbioinformatics.org). To use gVCF functionality and increased accuracy, genotyping was performed using GATK HaplotypeCaller, rather than UnifiedGenotyper. Variants with the following values were removed: “ReadPosRankSum < -2.0”, “MQRankSum < -2.0”, “QUAL < 30.0”, “QD < 3.0”, “FS > 30.0”, “MQ < 30.0”, “DP < 10”, and “GQ < 20.0”.

### 2.3. Variant Discovery and Filtering

Overall, we found thousands of de novo mutations in the 51 Korean families. When conducting the exome sequencing in the first stage (2012), not enough databases were available to perform comparison at that time. Therefore, we used more stringent parameters by in silico prediction, such as phyloP, SIFT, and MutationAssessor. Large-scale population databases, such as dbSNP, 1000genomes, ESP6500, ExAc, GME, HRCR, and Kaviar, were available at ANNOVAR website. Moreover, in silico prediction methods such as CADD, M-CAP, REVEL, FATHMM, and EIGEN, are available. Some of these databases provide scores for non-genic regions and coding regions. In the second stage, we used a series of filtering steps to select the most promising variants. First, we removed common variants at 1% frequency from all population databases, which were annotated within dbSNP along with sex chromosome variants (Supplementary Table S2). Eighteen dbSNP variants had small minor allele frequencies. Two known de novo mutations, rs778416774 and rs267606749, were identified as likely pathogenic in Human Genome Mutation Database (HGMD^®^) (dominant, proband; heterozygous) and pathogenic in ClinVar and HGMD^®^ (recessive, proband; heterozygous). Subsequently, we removed the variants from a list of de novo variants: (1) MODIFIER effect in snpEff, (2) variants onto simple repeats and low complexity regions, (3) low coverage area, and (4) LB and B classes in the InterVar database. Considering the long research periods, we have converted genomic coordinates into the GRCh38/hg38 genome and re-checked population frequencies using dbSNP, gnomAD exome v2.1.1, genome v3.0, and variant pathogenicities provided with commercial HGMD 2019.1 release and ClinVar August 2020 updates in NCBI. These two databases demonstrate an issue of false positives, as these records originate from various sources that are sometimes not fully verified.

### 2.4. Gene Network Analysis

Genetic networks of the 36 genes with de novo variants with known ASD-related genes were constructed with closely interacting gene neighbors using “variant effect” core analysis in the Ingenuity Pathway Analysis software (IPA, Qiagen). The purpose of “Interaction Network Analysis” is to construct connectivity map among genes based on transcriptional networks, microRNA-mRNA target networks, phosphorylation cascades, and protein–protein/protein–DNA interaction networks. The related networks were ranked according to their biological relevance in the provided gene list. Six genetic networks were generated using 36 genes with de novo variants with 49 known ASD-related genes. Significant associations between a gene set and canonical pathways were determined using the ratio of the number of focus genes that mapped to a canonical pathway divided by the total number of genes from the IPA that map to that pathway. The pathway analyses allow identifying biologically relevant genes compared to a single variant or gene–disease association tests. Furthermore, the software identifies top functions and diseases associated with each network, thereby highlighting the biological significance of the results.

The topologically associating domain (TAD) between the genes in hippocampus tissue were adopted from 3DIV website (http://3div.kr). It is derived from Hi-C sequencing for chromatin interaction and usually smaller than linage disequilibrium (LD) block. The statistically significant TADs are represented by red squares, while the low non-significant TADs are represented by white squares. The associated genes are grouped into a blue block.

### 2.5. Genetic Validation using Sanger Sequencing

Genomic DNA was extracted using the DNeasy Blood and Tissue Kit (Qiagen, USA). We designed PCR primers to validate the selected 36 de novo variants for each candidate site using the Primer3 program. Sanger sequencing was performed on an ABI 3730 sequencer to validate new de novo mutation candidates. Others were assayed using the Sequenom MassARRAY system following the manufacturer’s recommendations (Sequenom, Inc., San Diego, CA, USA). Finally, all candidates were validated using Sanger sequencing (Supplementary Figure S1).

## 3. Results

A total of 12 family trios and one patient (37 individuals) participated in the first stage, and 38 family trios (114 individuals) participated in the second stage. Four probands were female, and the mean age of the probands was 70.16 months (SD 27.35), while their mean full-scale IQ was 53.70 (SD 21.90). Table 1 summarized the clinical characteristics of the probands.

We identified each de novo variant from patients with ASD that was not found in other unrelated patients. In total, 36 de novo mutations in 26 probands were identified, with 27 missense, two silent, one nonsense, one splice region, one splice site, one 5′ UTR, one intronic SNVs, and two frameshift deletions (Table 2). Seven probands—B25 four de novo variants, B26 three de novo variants, and A12/B10/B21/B32/B5 two de novo variants—have more than one de novo variant as described at the last column. Also, we could not find any de novo variants in 27 probands. Five de novo variants (5’ UTR in *MTUS1*, antisense *PITRM1-AS1*/intronic *PITRM1*, silent in *SYNM* and *MT-ND2*, and splice region in *SNF8*) are not usual protein-altering variants, but those were included in the analysis as they were rare, with putative causal effects. *MTUS1* variants (rs377560516) were in the 5′ UTR region, and featured histone acetylation (H3K27Ac) peaks (often found near active regulatory elements), DNAseI hypersensitivity regions, and transcription factor POLR2A-binding motifs. Variant chr10 g.3141725G>A was found in the intronic region of *PITRM1*, as well as in the exonic region of *PITRM1-AS1*, which is an antisense gene. We found that the silent mutation p.Ser1099Ser of *SYNM* is in the DNAase I hypersensitivity region, which has been found once previously without any special annotations according to the gnomAD exome v2.1.1 database. The intronic variant of the *SNF8* was found in the deep intronic region near the 3′ donor site, but found no special features.

**Figure 2:**
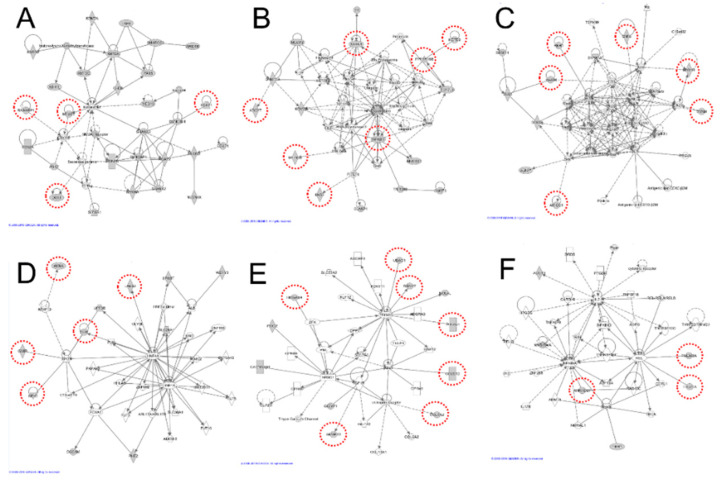
Novel autism spectrum disorder (ASD) risk genes with de novo variants in the gene network. Gene network (**A**–**F**) ASD risk genes with de novo variants in this study are circled in red and positioned into six gene networks. A: *DTX1*, *MTUS1*, *RASGRP1*, and *RUFY1*. B: *ADCY7*, *ATXN1*, *IWS1*, *KCTD9*, *MT-ND2*, *NFKB1*, and *PPP1R16B*. C: *AMIGO1*, *HAX1*, *MYH14*, *RNH1*, *SNF8* and *SYNM*. D: *AKNA*, *GMIP*, *LARS2*, *NEK1*, and *TFPT*. E: *CELSR3*, *COL6A2*, *HMGXB4*, *PIEZO1*, *RBM27*, *SFMBT2*, and *UBAC1*. F: *ANKRD27*, *TMEM8A*, and *TTC21A*.

The 36 de novo variants discovered were confirmed using Sanger sequencing, and the interaction networks of IPA identified the relationships between genes. Many of the identified ASD-risk genes were related to gene networks. The canonical pathways have shown many signaling interactions that contain two genes with de novo variants (*ADCY7* and *NFKB1*) (Supplementary Table S3). These genes also exhibit several neurological functions such as neurotransmission, memory, morphology of the nervous system, and neuronal development. These genes were also related to developmental or neurological diseases such as abnormal development of the central nervous system, brain cancer, abnormal development of neurons, and brain morphology (Supplementary Table S4). In particular, *ATXN1*, *NFKB1*, and *CELSR3* were abundantly found in these categories. Associated diseases and functions of these three genes involves developmental disorders, hereditary disorders, and neurological diseases (Supplementary Table S5). Our extended gene network revealed more gene interactions. The candidate causal ASD genes identified here are described by their functions and relationships with neurological diseases and functions. The gene network of discovered genes with de novo variants was constructed among the significant molecular networks from commercial knowledge, which allows the connection of known neurological functional genes, and thereby building strong evidence of the neurological roles within the six gene networks (Figure 2).

### 3.1. Network A. DTX1, MTUS1, RASGRP1, and RUFY1

Four candidate genes interacting with 24 known ASD-related genes were included in the gene network A (Figure 2A, Supplementary Table S5). Among these genes, a novel de novo mutation in *RUFY1* (RUN and FYVE Domain Containing 1) is believed to bind with DYNC1H1 by protein–protein interactions. A de novo mutation of *DYNC1H1* [28] associated with epileptic encephalopathy [29] was found to be shared among different neuropsychiatric disorders. *RUFY1* is also related to metabolism and endocytosis pathways, which is ubiquitously expressed in various tissues [30].

### 3.2. Network B. ADCY7, ATXN1, IWS1, KCTD9, MT-ND2, NFKB1, and PPP1R16B

In gene network B, the network was constructed using seven candidate genes and nine known ASD-related genes (Figure 2B, Supplementary Table S5). The genes involved in this network have protein–protein interactions that are connected with the NF-κB signaling pathway. The key gene of this network is *NFKB1*, which is strongly associated with disease related to immunodeficiency. Rett syndrome phenotypes presented in MECP2-null mice indicate the key role of NF-κB signaling in RTT pathogenesis [31]. In addition, gastrointestinal dysfunction symptoms associated with autism include *NFKB1* as a biomarker [32]. The interference of *NFKB1* by siRNA decreases the expression of ANKRD1 in cultured human aortic smooth muscle cells (HASMC). *ANKRD1* encodes a nuclear protein and functions in global transcriptional regulation, and plays a role in the positioning and early growth of neurons in the brain [33]. In particular, *NFKB1* exhibited many connected canonical pathways, indicating that the interactions are strongly connected to other proteins related to the development and function of the nervous system (Supplementary Table S3).

ATXN1 is associated with spinocerebellar ataxia 1 and spinocerebellar degeneration by affecting AKT signaling and checkpoint regulation [34]. ATXN1 is involved in the transcriptional repression of the CIC (Capicu transcriptional repressor) protein, and de novo mutations in CIC have been reported to occur in intellectual disabilities, attention deficit/hyperactivity disorder (ADHD), and ASD [35]. Moreover, ATXN1 binds CIC to form a protein complex, and the disruption of this complex causes neurobehavioral phenotypes in humans and mice [36]. ADCY7 interacts with salivary secretion and DAG/IP3 signaling pathways, and encodes a membrane-bound adenylate cyclase that catalyzes the formation of cyclic AMP from ATP, which is inhibitable by calcium. Although DNMT3A is involved in de novo DNA methylation and mutation processes that can cause overgrowth syndrome with intellectual disabilities, the interference of mouse *DNMT3A* decreases the expression of mouse ADCY7 in cardiomyocytes from embryonic mice, suggesting that interactions exist between ADCY7 and DNMT3A [35]. In mouse oligodendrocyte progenitor cells, knockout of *TCFL2* decreases expression of PPP1R16B in the spinal cord, and de novo mutations in *TCF7L2* are involved in ASD [28]. Furthermore, *PPP1R16B* has a central role in the integration of fast and slow neurotransmission in schizophrenia [37].

### 3.3. Network C. AMIGO1, HAX1, MYH14, RNH1, SNF8, and SYNM

The gene network C was constructed using six candidate genes and six known ASD-related genes (Figure 2C, Supplementary Table S5). Among them, two genes showed associations with neurological disorders. RNH1 binds to PTEN in the LN229 cell lysate, while *PTEN* mutation is associated with ASD, as confirmed in an independent study [38,39]. Moreover, previous studies have reported that patients with 11p15.5 and 20q13.33 deletions, which include mutations in *RNH1*, had conduct disorder (CD), ADHD, and intellectual disabilities [40]. The HAX1 protein binds to CUL3, and de novo mutations of *CUL3* have been reported the relationship to WNT signaling and chromatin regulation [41]. Roberts et al. reported that CMA (chromosomal microarray analysis) revealed an association between ASD and a 1q21.3-q23.1 deletion, where the genomic position of HAX1 gene is [42].

### 3.4. Network D. AKNA, GMIP, LARS2, NEK1, and TFPT

The gene network D was constructed with five genes and five known ASD-related genes (Figure 2D, Supplementary Table S5). GMIP, NEK1, and TFPT are involved in protein interactions with XPO1. Previous research has shown that *XPO1* (rs6735330) is significantly associated with ASD in family-based studies [43]. This allele is significantly associated with more severe deficits in social interaction, verbal communication, and repetitive behaviors. Therefore, such findings suggest that this allele is in high LD with a functional polymorphism and a mutation in this gene may serve as a risk factor for ASD.

### 3.5. Network E. CELSR3, COL6A2, HMGXB4, PIEZO1, RBM27, SFMBT2, and UBAC1

Seven candidate genes and three known ASD-related genes were included in the gene network E (Figure 2E, Supplementary Table S5). Among these genes, *CELSR3* is known to be involved in childhood neurodevelopmental mental health issues [44]. Moreover, mutations in *CELSR3* were also identified in 51 patients with ASD and their families [45]. We discovered a total of 109 de novo variants, where 29 variants were found to be deleterious or potentially causing deleterious effects, including *CELSR3*, which can affect the central nervous system.

### 3.6. Network F. ANKRD27, TMEM8A, and TTC21A

The gene network F was constructed using three genes and two known ASD-related genes (Figure 2F, Supplementary Table S5). Rare copy number variations in ASD family studies include genes that are primarily associated with ASD. The genetic association analysis in the Korean subjects identified loss of heterozygosity (LOHs) with the risk of Hirschsprung (HSCR) disease in *TTC21A* [46].

### 3.7. Evidence from Topologically Associating Domain (TAD) in TFPT and HAX1 

**Figure 3:**
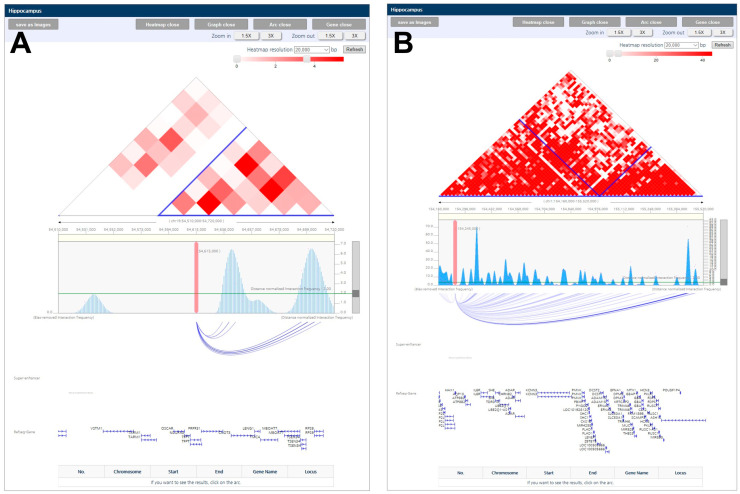
De novo variant genes associated with neurological functional genes in the TAD. (**A**) *TFPT* is associated with CNOT3 in the same TAD. (**B**) *HAX1* is associated with ASH1L in the same TAD.

Some of the de novo variants identified were associated with ASD or neurological diseases within the TAD, which were associated with genes that significantly contribute to ASD (Figure 3). *TFPT*, as observed in Figure 3A, was contained within *CNOT3* in the same TAD. A recent GWAS using whole genome sequencing in 5205 patients confirmed that 18 candidate common genes, including *CNOT3*, are associated with ASD [47]. As observed in Figure 3B, *HAX1* was contained within *ASH1L* in the same TAD, as well as the gene network in Figure 2C. A total of 189 risk genes were sequenced using single-molecule molecular inversion probes in 1543 Chinese ASD probands, and one of the prevalent genes identified with recurrent de novo mutation was *ASH1L*. Furthermore, 208 candidate genes from 11,730 cases were identified with *ASH1L* by clinical follow-up of the syndromic and non-syndromic forms of the disease [48,49]. This result suggests that the two genes are strongly associated with ASD, as supported by previous studies.

## 4. Discussion

In this study, we performed exome sequencing to identify putative causal variants of ASD in 51 Korean families, and identified 36 de novo variants. We identified putative functional relationships with ASD by interaction network analysis, but our findings need to be validated by further functional studies, possibly with other neurological diseases. All variants were successfully validated using Sanger sequencing. Genetic network analyses showed that the variants confirmed in this study are located in six different networks. Previous studies reported that these networks are functionally related to neuronal development and various neurological or psychiatric conditions, including epileptic encephalopathy, Rett syndrome, schizophrenia, intellectual disability, and ASD. For example, in network A, *RUFY1* showed interactions with the neuropsychiatric disorder-related gene *DYNC1H1* [28]. *RNH1* from network C was associated with *PTEN*, which is a causative gene of ASD [50]. Other genes, such as *GMIP*, *NEK1*, *TFPT*, *CELSR3*, and *TTC21A*, also showed interactions with ASD or other neurodevelopmental disorder-related genes in each gene network in the previous studies [38,39,40,41,42]. We can infer that pathophysiological mechanisms derived from those genes or genetic networks is partially shared with ASD and other developmental disturbances of central nervous system. Overall, interaction maps from the novel candidate genes with known ASD-related genes suggested that our novel de novo variants are strong candidates of risk genes for ASD.

We found novel de novo variants that might be functionally linked to ASD, which are particularly related to functional genetic networks such as the NF-κB signaling pathway. Among the interactions between the causal ASD de novo variants in this study, NF-κB signaling-related genes are the most noteworthy, as those genes were commonly observed in Network B (*NFKB1*) and F (*NFKB1A* and *NFKB1D*) based on the IPA assay results (Figure 2). Moreover, some individuals with ASD suffer from other neurological or physical illnesses such as immune dysfunction, which may be induced by variable associated genes related to several signal transduction such as NF-kβ [51]. NF-κB is a transcription factor involved in cellular immune and inflammatory responses, development, cell proliferation, and apoptosis [52]. In addition, the NF-κB signaling pathway is an essential regulator in the development and maturation of the nervous system [53]. During neurogenesis, NF-κB signaling mediates the effect of several biological factors such as cytokines and growth factors, and facilitates crosstalk with other signaling pathways such as NOTCH, SHH, and WNT/β-catenin [54]. Furthermore, several studies have reported that NF-κB plays an important role in controlling axon and dendrite growth during development and dendritic spine density in adults [55]. A previous study found that the NF-κB transcription factor family is regulated by group I metabotropic glutamate receptors in the hippocampus and the c-Rel transcription factor is necessary for long-term maintenance of long-term depression (LTD) in the cells and formation of long-term memory [56]. It was recently demonstrated that NF-κB is important in synaptic plasticity as well as in learning and memory. Thus far, a relatively small number of NF-κB target genes have been found in neurons, including Ca^2+^-calmodulin-dependent protein kinase II (CaMKII) δ, brain-derived neurotrophic factor, μ-opioid receptors, neural cell adhesion molecule, inducible nitric oxide synthetase, and amyloid precursor protein [57]. It is also well known that NF-κB is a pro-inflammatory pathway and its activation is responsible for elevated inflammatory cytokines. In the CNS, NF-κB-mediated transcriptional control regulates hundreds of genes including the inflammatory responses such as microglia activation, which potentially affects the brain homeostasis, neural connectivity, and thus behavior [58].

We can potentially attribute the complex clinical phenotypes that characterize ASD to the NF-κB signaling, given the essential functions it plays in the mechanisms of brain developments. In studies with animal models, abnormal NF-κB signaling has been frequently reported in valproic acid (VPA)-exposed models of ASD. These include elevated p65 expression after treatment with VPA in human neuroblastoma cell line SHSY-5Y, which provides evidence that VPA affects the NF-κB pathway [59]. Another study reported that VPA inhibits neural progenitor cell death by activating the NF-κB signaling pathway, which subsequently enhances the expression of the antiapoptotic protein Bcl-XL [60]. Kishi et al. (2016) reported that genetically reducing NF-κB signaling in Mecp2-null mice not only ameliorated cortical callosal projection neurons and dendritic complexity, but also substantially extended their normally shortened lifespan [31]. In studies with human samples, several reports indicate that NF-κB concentrations were elevated in the serum of subjects with ASD and postmortem samples of orbitofrontal cortex tissues [61,62].

With these findings, it is suggested that NF-κB-related immune responses are altered in the brain and periphery in the context of ASD [63]. Though more studies correlating neuroinflammation and immune cell function, within NF-κB and cytokine signaling, are needed to broaden our understanding of ASD-related immunopathology, NF-κB may be governing a broad range of neurological features such as neuronal development, homeostasis, as well as immune regulation in ASD [64]. Although it is unknown whether NF-κB acts as a significant cause of ASD or regulates the onset of ASD as a cofactor, the evidence generated in both human studies and animal models shows the influence NF-κB in the neurobiological basis of ASD [65].

ASD is characterized by complex clinical phenotypes, it would be worth analyzing the generic network according to the subjects’ symptom profiles. Considering the relatively high mean in the ADI-R domain scores and having ADOS modules 1 or 2 administered to 98% of the subjects, it could be assumed that the NF-κB pathway might be associated with a specific subset of ASD, especially those with typical, severe phenotypes. However, the nine probands in our samples who have rare de novo variants in the genes interacting with the NF-κB signaling pathway did not show significant differences in the algorithm scores of ADI-R, as well as IQ. Further replication studies would be needed to evaluate quantitative relationships of NF-κB-related rare variants and specific phenotypes of ASD, especially those with fluent language and/or normal range of IQ.

### Limitations

There are some limitations of this study. The relatively small size may affect the results of novel ASD genes with de novo variants. We specifically searched for rare variants in a small Korean cohort, and identifying variants using MAF filtering can result in false negatives due to the small sample size. Moreover, we did not conduct a functional experiment; therefore, distinguishing between rare nonfunctional and rare functional variants is not feasible. In order to show a probable relationship with known autism-related genes, we performed network analysis using IPA and demonstrated that some of the de novo variants could be related. Due to the small sample size, we were unable to include the subgroup analyses according to ASD symptom severity. Though we demonstrate a possible signaling pathway (NF-κB) with the de novo variants in the Korean individuals with ASD, we could not suggest the direct genetic causes by de novo variants. There is recent speculation of genetic association between an SNP in *NFKBIL1* and autism-like traits [66]. However, there is still a lack of studies looking at the genetic association between autism and NF-κB related genes. Further genetic association studies with more variants and populations are needed.

## 5. Conclusions

To identify putative causal rare genetic variants involved in ASD, we performed exome sequencing in 51 Korean families with ASD. We identified 36 novel de novo genetic variants. Interaction network analyses showed six different networks, possibly associated with neurological development or functions, as well as various neurodevelopmental or psychiatric conditions. The characterization of the network highlighted by the potential role of NF-κB signaling pathway in the pathogenesis of ASD.

## Figures and Tables

**Table 1:** Clinical characteristics of probands.

Clinical Characteristics		First Stage (*n* = 13)		Second Stage (*n* = 38)	
		Mean	SD	Mean	SD
Age (months)		59.50	12.41	73.53	29.94
Sex (number of females)		0		4	
ADI-R	social interaction	24.33	3.65	22.05	5.67
	communication: verbal	18.43	5.68	18.58	4.50
	communication: nonverbal	12.00	2.73	11.71	2.34
	repetitive behavior	6.50	2.71	5.74	2.58
ADOS	Communication	6.67	1.23	6.05	1.23
	social interaction	10.25	2.34	10.16	2.15
	Play	2.92	1.24	2.34	1.17
	repetitive behavior	3.00	1.65	2.50	1.39
KEDI-WISC-R		43.00	33.41	57.47	15.88
K-Leiter-R		78.86	25.60	62.56	25.64
SCQ		23.75	5.55	19.97	7.71
SRS		109.08	27.62	95.08	31.05

Abbreviations: ADI-R: Autism Diagnostic Interview—Revised; ADOS: Autism Diagnostic Observation Schedule; KEDI-WISC-R: Korean Educational Development Institute—Wechsler Intelligence Scale for Children—Revised; K-Leiter-R: Korean-Leiter International Performance scale—Revised; SCQ: Social Communication Questionnaire; SRS: Social Responsiveness Scale.

**Table 2:** De novo mutations observed in 51 Korean ASD trios.

Genomic Position	Gene Symbol	dbSNP RS ID	REF>ALT Allele	Sequence Variant Nomenclature ^(2)^	Variant Type	Probands (:occur.)
(GRCh38/hg38) ^(1)^		Build 154				
chr1:109507558	AMIGO1		C>A	ENST00000369864.5 c.1355G>T/p.Gly452Val	Missense	B34
chr1:154274980	HAX1		C>A	ENST00000328703.11|ENST00000457918.6 c.535C>A|c.391C>A/p.Pro179Thr|p.Pro131Thr	Missense	B15
chr2:127493330	IWS1		C>G	ENST00000295321.9|ENST00000637187.1 c.1880G>C|c.47G>C/p.Ser627Thr|p.Ser16Thr	Missense	B5 :1st
chr3:39112529	TTC21A		C>A	ENST00000431162.6 c.507C>A/p.Tyr169 *	Nonsense	B16
chr3:45513187	LARS2	rs147745374	C>T	ENST00000415258.6 c.1813C>T/p.Arg605Cys	Missense	B1
chr3:48645607	CELSR3	rs756781285	C>T	ENST00000164024.5 c.7633G>A/p.Val2545Met	Missense	A12 :1st
chr4:990079	SLC26A1	rs748023701	C>T	ENST00000361661.6 c.860G>A/p.Arg287His	Missense	B27
chr4:102607251	NFKB1	rs934744465	G>A	ENST00000394820.8 c.2053G>A/p.Gly685Arg	Missense	B25 :1st
chr4:169602542	NEK1	rs200825809	T>C	ENST00000507142.5 c.89A>G/p.Tyr30Cys	Missense	B11
chr5:146233644	RBM27		C>A	ENST00000265271.7 c.1045C>A/p.Pro349Thr	Missense	B26 :1st
chr5:179609380	RUFY1		A>G	ENST00000393438.6 c.1664A>G/p.His555Arg	Missense	B29
chr6:16306637	ATXN1		C>T	ENST00000244769.8 c.2140G>A/p.Asp714Asn	Missense	B26 :2nd
chr6:44282283	TCTE1	rs746423492	C>T	ENST00000371505.5 c.1123G>A/p.Glu375Lys	Missense	A4
chr8:17697396	MTUS1	rs377560516	G>A	ENST00000297488.10 c.-19C>T/5’UTR	5’UTR	B26 :3rd
chr8:25436242	KCTD9	rs1448423271	C>T	ENST00000221200.9 c.656G>A/p.Arg219Gln	Missense	A9
chr9:114342063	AKNA		A>T	ENST00000307564.8 c.3820T>A/p.Cys1274Ser	Missense	A12 :2nd
chr9:135939739	UBAC1		C>A	ENST00000371756.3 c.897G>T/p.Glu299Asp	Missense	B10 :1st
chr10:3141725	PITRM1-AS1		G>A	ENST00000430356.3 (PITRM1-AS1, Antisense) ENST00000451454.5 (PITRM1, Intronic)	Antisense/Intronic	B25 :2nd
chr10:7370280	SFMBT2	rs1373335289	C>T	ENST00000361972.8 c.195+1G>A/Splice Donor Variant	Splice Site	B5 :2nd
chr11:499976	RNH1		C>T	ENST00000534797.5 c.296G>A/p.Gly99Glu	Missense	B33
chr12:113094062	DTX1	rs1232450939	G>A	ENST00000257600.3 c.1190G>A/p.Arg397Gln	Missense	B6
chr15:38516208	RASGRP1	rs1595848108	G>A	ENST00000310803.10 c.664C>T/p.Arg222Trp	Missense	B12
chr15:99131660	SYNM	rs782771735	G>A	ENST00000336292.10 c.3300G>A/p.Ser1100Ser	Silent	B18
chr16:377706	TMEM8A		C>G	ENST00000431232.7 c.264G>C/p.Glu88Asp	Missense	B25 :3rd
chr16:50293480	ADCY7	rs199730202	G>A	ENST00000254235.7 c.814G>A/p.Val272Ile	Missense	B10 :2nd
chr16:88738626	PIEZO1	rs1475582880	GGCCGTGACTCGGAAACGAGCGGCCA>G	ENST00000301015.14 c.551_575delTGGCCGCTCGTTTCCGAGTCACGGC/p.Leu184fs	Frameshift Deletion	B3
chr17:48937128	SNF8		C>T	ENST00000502492.5 c.245-4G>A/Spice Region Variant	Splice Region	B25 :4th
chr19:19637386	GMIP		T>A	ENST00000203556.9 c.1103A>T/p.Glu368Val	Missense	B21 :1st
chr19:32628120	ANKRD27		G>C	ENST00000306065.9 c.1383C>G/p.Asp461Glu	Missense	B21 :2nd
chr19:50257343	MYH14	rs778416774	G>A	ENST00000599920.5 c.1990G>A/p.Gly664Ser	Missense	A3
chr19:54107093	TFPT		TTGTC>T	ENST00000391759.5 c.715_718delGACA/p.Asp239fs	Frameshift Deletion	B4
chr20:32453760	NOL4L	rs749152593	G>C	ENST00000359676.9 c.389C>G/p.Ser130Cys	Missense	B9
chr20:38902679	PPP1R16B		G>A	ENST00000299824.6 c.583G>A/p.Glu195Lys	Missense	B7
chr21:46121590	COL6A2	rs267606749	G>A	ENST00000300527.8 c.1493G>A/p.Arg498His	Missense	B37
chr22:35265292	HMGXB4		C>G	ENST00000455359.5 c.577C>G/p.Leu193Val	Missense	B32 :1st
chrM:4943	MT-ND2	rs1603219681	A>G	ENST00000361453.3 c.474A>G/p.Ser158Ser	Silent	B32 :2nd

^(1)^ Variants discovered onto GRCh37/hg19 assembly, but converted toGRCh38/hg38 assembly by liftOver software. ^(2)^ Gene annotation is done by snpEff software using GENCODE v30 primary transcripts, *: stop codon.

## Data Availability

The raw datasets generated or analyzed during the current study are not publicly available because the public access was not consented from the participants in the study, but are available from the corresponding author on reasonable request and materials.

## Supplementary Materials

The following are available online at https://www.mdpi.com/2073-4425/12/1/1/s1, Table S1: Data summary for exome data. Table S2: Known dbSNP rsID (build 154) and minor allele frequency (MAF). Table S3: Genes connected with canonical pathways by IPA. Table S4: Genes related to neurological function or disease. Table S5: Top diseases and functions from 36 candidate ASD gene network. Figure S1: Sanger validation of *de novo* variants.

## Abbreviations

ASD: Autism Spectrum Disorder, GWAS: Genome-Wide Association Studies, NGS: Next Generation Sequencing, IPA: Ingenuity Pathway Analysis, GATK: Genome Analysis Toolkit, ACMG/AMP: American College of Medical Genetics and Genomics/Association for Molecular Pathology, HGMD: Human Gene Mutation Database, TAD: Topologically Associating Domain, LD: Linkage Disequilibrium, WES: Whole Exome Sequencing, UTR: Untranslated region, SNV: Single-Nucleotide Variation, SNP: Single-Nucleotide Polymorphism, GATK: Genome Analysis Tool Kit (https://gatk.broadinstitute.org).

## References

[1] Yoo H.J., Kim B.-N., Kim J.-W., Shin M.-S., Park T.-W., Son J.-W., Chung U.-S., Park M., Kim S.A. (2017). Family-based genetic association study of CNTNAP2 polymorphisms and sociality endophenotypes in Korean patients with autism spectrum disorders. Psychiatr. Genet..

[2] Woodbury-Smith M., Scherer S.W. (2018). Progress in the genetics of autism spectrum disorder. Dev. Med. Child Neurol..

[3] Ghirardi L., Brikell I., Kuja-Halkola R., Freitag C.M., Franke B., Asherson P., Lichtenstein P., Larsson H. (2018). The familial co-aggregation of ASD and ADHD: A register-based cohort study. Mol. Psychiatry.

[4] Mitchell K.J. (2011). The genetics of neurodevelopmental disease. Curr. Opin. Neurobiol..

[5] Chahrour M.H., Yu T.W., Lim E.T., Ataman B., Coulter M.E., Hill R.S., Stevens C.R., Schubert C.R., Greenberg M.E., Gabriel S.B. (2012). Whole-Exome Sequencing and Homozygosity Analysis Implicate Depolarization-Regulated Neuronal Genes in Autism. PLoS Genet..

[6] Elia J., Gai X., Xie H.M., Perin J.C., Geiger E., Glessner J.T., Darcy M., Deberardinis R., Frackelton E., Kim C. (2009). Rare structural variants found in attention-deficit hyperactivity disorder are preferentially associated with neurodevelopmental genes. Mol. Psychiatry.

[7] González A.A., Cabanas M.C., Rodriguez Fontenla M.C., Carracedo A. (2019). Novel gene-based analysis of ASD GWAS: Insight into the biological role of associated genes. Front. Genet..

[8] Robinson E.B., Pourcain B.S., Anttila V., Kosmicki J., Bulik-Sullivan B., Grove J., Maller J., Samocha K.E., Sanders S., Ripke S. (2016). Genetic risk for autism spectrum disorders and neuropsychiatric variation in the general population. Nat. Genet..

[9] Autism Spectrum Disorders Working Group of the Psychiatric Genomics Consortium (2017). Meta-analysis of GWAS of over 16,000 individuals with autism spectrum disorder highlights a novel locus at 10q24.32 and a significant overlap with schizophrenia. Mol. Autism..

[10] Sztainberg Y., Zoghbi H.Y. (2016). Lessons learned from studying syndromic autism spectrum disorders. Nat. Neurosci..

[11] Fernandez B.A., Scherer S.W. (2017). Syndromic autism spectrum disorders: Moving from a clinically defined to a molecularly defined approach. Dialogues Clin. Neurosci..

[12] Nakanishi M., Nomura J., Ji X., Tamada K., Arai T., Takahashi E., Bućan M., Takumi T. (2017). Functional significance of rare neuroligin 1 variants found in autism. PLoS Genet..

[13] Rafiq M.A., Leblond C.S., Saqib M.A.N., Vincent A.K., Ambalavanan A., Khan F.S., Ayaz M., Shaheen N., Spiegelman D., Ali G. (2015). Novel VPS13B Mutations in Three Large Pakistani Cohen Syndrome Families Suggests a Baloch Variant with Autistic-Like Features. BMC Med. Genet..

[14] Delahanty R.J., Kang J., Brune C.W., Kistner E.O., Courchesne E., Cox N.J., Cook E.H., Macdonald R.L., Sutcliffe J.S. (2011). Maternal transmission of a rare GABRB3 signal peptide variant is associated with autism. Mol. Psychiatry.

[15] Torrico B., Fernàndez-Castillo N., Hervás A., Milà M., Salgado M., Rueda I., Buitelaar J.K., Rommelse N., Oerlemans A.M., Bralten J. (2015). Contribution of common and rare variants of the PTCHD1 gene to autism spectrum disorders and intellectual disability. Eur. J. Hum. Genet..

[16] Schaaf C.P., Zoghbi H.Y. (2011). Solving the Autism Puzzle a Few Pieces at a Time. Neuron.

[17] Weiner D.J., Wigdor E.M., Ripke S., Walters R.K., Kosmicki J.A., Grove J., Samocha K.E., Goldstein J.I., Okbay A., Bybjerg-Grauholm J. (2017). Polygenic transmission disequilibrium confirms that common and rare variation act additively to create risk for autism spectrum disorders. Nat. Genet..

[18] Hazlett H.C., Gu H., Munsell B.C., Kim S.H., Styner M., Wolff J.J., Elison J.T., Swanson M.R., Zhu H., Botteron K.N. (2017). Early brain development in infants at high risk for autism spectrum disorder. Nature.

[19] Feliciano P., Zhou X., Astrovskaya I., Turner T.N., Wang T., Brueggeman L., Barnard R., Hsieh A., Snyder L.G., Muzny D.M. (2019). Exome sequencing of 457 autism families recruited online provides evidence for autism risk genes. NPJ Genom. Med..

[20] Lee J., Ha S., Lee S.T., Park S.G., Shin S., Choi J.R., Cheon K.A. (2020). Next-Generation Sequencing in Korean Children With Autism Spectrum Disorder and Comorbid Epilepsy. Front. Pharmacol..

[21] Stein D.J., Phillips K.A. (2013). Patient advocacy and DSM-5. BMC Med..

[22] Lord C., Rutter M., Le Couteur A. (1994). Autism Diagnostic Interview-Revised: A revised version of a diagnostic interview for caregivers of individuals with possible pervasive developmental disorders. J. Autism Dev. Disord..

[23] Lord C., Risi S., Lambrecht L., Cook E.H., Leventhal B.L., DiLavore P.C., Pickles A., Rutter M. (2000). The Autism Diagnostic Observation Schedule—Generic: A standard measure of social and communication deficits associated with the spectrum of autism. J. Autism Dev. Disord..

[24] Park K., Yoon J., Park H., Kwon K. (2002). Korean Educational Developmental Institute-Wechsler Intelligence Scale for Children (KEDI-WISC).

[25] Shin M., Cho S. (2010). Korean Leiter International Performance Scale-Revised.

[26] Rutter M., Bailey A., Lord C. (2003). The Social Communication Questionnaire: Manual.

[27] Constantino J.N., Gruber C.P. (2012). Social Responsiveness Scale: SRS-2 Software Kit.

[28] Iossifov I., O’roak B.J., Sanders S.J., Ronemus M., Krumm N., Levy D., Stessman H.A., Witherspoon K.T., Vives L., Patterson K.E. (2014). The contribution of de novo coding mutations to autism spectrum disorder. Nature.

[29] Lin Z., Liu Z., Li X., Li F., Hu Y., Chen B., Wang Z., Liu Y. (2017). Whole-exome sequencing identifies a novel de novo mutation in DYNC1H1 in epileptic encephalopathies. Sci. Rep..

[30] Seet L.-F., Hong W. (2001). Endofin, an Endosomal FYVE Domain Protein. J. Biol. Chem..

[31] Kishi N., MacDonald J.L., Ye J., Molyneaux B.J., Azim E., Macklis J.D. (2016). Reduction of aberrant NF-κB signalling ameliorates Rett syndrome phenotypes in Mecp2-null mice. Nat. Commun..

[32] Shindler A.E., Hill-Yardin E.L., Petrovski S., Bishop N., Franks A.E. (2019). Towards Identifying Genetic Biomarkers for Gastrointestinal Dysfunction in Autism. J. Autism Dev. Disord..

[33] Li H., Wang Z., Ma T., Wei G., Ni T. (2017). Alternative splicing in aging and age-related diseases. Transl. Med. Aging.

[34] Ingram M., Wozniak E.A., Duvick L., Yang R., Bergmann P., Carson R., O’Callaghan B., Zoghbi H.Y., Henzler C., Orr H.T. (2016). Cerebellar Transcriptome Profiles of ATXN1 Transgenic Mice Reveal SCA1 Disease Progression and Protection Pathways. Neuron.

[35] Esteban F.J., Wall D.P. (2011). Using game theory to detect genes involved in Autism Spectrum Disorder. Top.

[36] Lu S., Zhao X., Hu Y., Liu S., Nan H., Li X., Fang C., Cao D., Shi X., Kong L. (2017). Natural variation at the soybean J locus improves adaptation to the tropics and enhances yield. Nat. Genet..

[37] Devor A., Andreassen O.A., Wang Y., Mäki-Marttunen T., Smeland O.B., Fan C.-C., Schork A.J., Holland D., Thompson W.K., Witoelar A. (2017). Genetic evidence for role of integration of fast and slow neurotransmission in schizophrenia. Mol. Psychiatry.

[38] Evans J.C., Archer H.L., Colley J.P., Ravn K., Nielsen J.B., Kerr A., Williams E., Christodoulou J., Gécz J., Jardine P.E. (2005). Early onset seizures and Rett-like features associated with mutations in CDKL5. Eur. J. Hum. Genet..

[39] Mcbride K.L., Varga E.A., Pastore M.T., Prior T.W., Manickam K., Atkin J.F., Herman G.E. (2010). Confirmation study of PTEN mutations among individuals with autism or developmental delays/mental retardation and macrocephaly. Autism Res..

[40] Kolaitis G., Bouwkamp C.G., Papakonstantinou A., Otheiti I., Belivanaki M., Haritaki S., Korpa T., Albani Z., Terzioglou E., Apostola P. (2016). A boy with conduct disorder (CD), attention deficit hyperactivity disorder (ADHD), borderline intellectual disability, and 47, XXY syndrome in combination with a 7q11.23 duplication, 11p15.5 deletion, and 20q13.33 deletion. Child Adolesc. Psychiatry Ment. Health.

[41] Shao L., Cui L., Lu J., Lang Y., Bottillo I., Zhao X. (2018). A novel mutation in exon 9 of Cullin 3 gene contributes to aberrant splicing in pseudohypoaldosteronism type II. FEBS Open Bio.

[42] Roberts J.L., Hovanes K., Dasouki M., Manzardo A.M., Butler M.G. (2014). Chromosomal microarray analysis of consecutive individuals with autism spectrum disorders or learning disability presenting for genetic services. Gene.

[43] Liu X., Malenfant P., Reesor C., Lee A., Hudson M.L., Harvard C., Qiao Y., Persico A.M., Cohen I.L., Chudley A.E. (2011). 2p15–p16.1 microdeletion syndrome: Molecular characterization and association of the OTX1 and XPO1 genes with autism spectrum disorders. Eur. J. Hum. Genet..

[44] Wilkes T., Wang E., Perry B. (2017). A neuro-developmentally sensitive and trauma informed service delivery approach for child and youth mental health and psychiatry. Eur. Psychiatry.

[45] Yoo H., Kim S., Park M., Kim J., Lim W., Noh D., Han D., Shin C., Kim N. (2017). Family-based Whole Exome Sequencing of Autism Spectrum Disorder Reveals Novel De Novo Variants in Korean Population. Eur. Psychiatry.

[46] Kim J.-H., Yoon K.-O., Kim J.-K., Kim J.-W., Lee S.-K., Kong S.-Y., Seo J.-M. (2006). Novel mutations of RET gene in Korean patients with sporadic Hirschsprungs disease. J. Pediatr. Surg..

[47] Yuen R.K., Merico D., Bookman M., Howe J.L., Thiruvahindrapuram B., Patel R.V., Whitney J., Deflaux N., Bingham J., Wang Z. (2017). Whole genome sequencing resource identifies 18 new candidate genes for autism spectrum disorder. Nat. Neurosci..

[48] Wang T., Guo H., Xiong B., Stessman H., Xia K., Eichler E. (2016). De Novo Genic Mutations Among A Chinese Autism Spectrum Disorder Cohort. Nat. Commun..

[49] Stessman H.A.F., Xiong B., Coe B.P., Wang T., Hoekzema K., Fenckova M., Kvarnung M., Gerdts J., Trinh S., Cosemans N. (2017). argeted sequencing identifies 91 neurodevelopmental-disorder risk genes with autism and developmental-disability biases. Nat. Genet..

[50] Kim Y., Park S., Choi E.Y., Kim S., Kwak H.J., Yoo B.C., Yoo H., Lee S., Kim D., Park J.B. (2011). PTEN modulates miR-21 processing via RNA-regulatory protein RNH1. PLoS ONE.

[51] Bjorklund G., Saad K., Chirumbolo S., Kern J.K., Geier D.A., Geier M.R., Urbina M.A. (2016). Immune dysfunction and neuroinflammation in autism spectrum disorder. Acta Neurobiol. Exp..

[52] Li N., Karin M. (1999). Is NF-κB the sensor of oxidative stress?. FASEB J..

[53] Gutierrez H., Davies A.M. (2011). Regulation of neural process growth, elaboration and structural plasticity by NF-κB. Trends Neurosci..

[54] Zhang Y., Hu W. (2012). NFκB signaling regulates embryonic and adult neurogenesis. Front. Biol..

[55] Roussos P., Katsel P., Davis K.L., Giakoumaki S.G., Siever L.J., Bitsios P., Haroutunian V. (2012). Convergent Findings for Abnormalities of the NF-κB Signaling Pathway in Schizophrenia. Neuropsychopharmacology.

[56] O’Riordan K.J., Huang I.C., Pizzi M., Spano P., Boroni F., Egli R., Desai P., Fitch O., Malone L., Ahn H.J. (2006). Regulation of Nuclear Factor B in the Hippocampus by Group I Metabotropic Glutamate Receptors. J. Neurosci..

[57] Alberini C.M. (2009). Transcription Factors in Long-Term Memory and Synaptic Plasticity. Physiol. Rev..

[58] Rodriguez J.I., Kern J.K. (2011). Evidence of microglial activation in autism and its possible role in brain underconnectivity. Neuron Glia Biol..

[59] Dodurga Y., Gundogdu G., Tekin V., Koc T., Satiroglu-Tufan N.L., Bagci G., Kucukatay V. (2014). Valproic acid inhibits the proliferation of SHSY5Y neuroblastoma cancer cells by downregulating URG4/URGCP and CCND1 gene expression. Mol. Biol. Rep..

[60] Go H., Seo J., Kim K., Han S., Kim P., Kang Y., Han S., Shin C., Ko K. (2011). Valproic acid inhibits neural progenitor cell death by activation of NF-κB signaling pathway and up-regulation of Bcl-XL. J. Biomed. Sci..

[61] Abdel-Salam O.M.E., Youness E.R., Mohammed N.A., Elhamed W.A.A. (2015). Nuclear Factor-Kappa B and Other Oxidative Stress Biomarkers in Serum of Autistic Children. Open J. Mol. Integr. Physiol..

[62] Young A.M.H., Campbell E., Lynch S., Suckling J., Powis S.J. (2011). Aberrant NF-KappaB Expression in Autism Spectrum Condition: A Mechanism for Neuroinflammation. Front. Psychiatry.

[63] Nadeem A., Ahmad S.F., Attia S.M., Bakheet S.A., Al-Harbi N.O., AL-Ayadhi L.Y. (2018). Activation of IL-17 receptor leads to increased oxidative inflammation in peripheral monocytes of autistic children. Brain Behav. Immun..

[64] Baranova J., Dragunas G., Botellho M.C.S., Ayub A.L.P., Bueno‑Alves R., Alencar R.R., Papaiz D.D., Sogayar M.C., Ulrich H., Correa R.G. (2020). Autism Spectrum Disorder: Signaling Pathways and Prospective Therapeutic Targets. Cell. Mol. Neurobiol..

[65] Liao X., Li Y. (2020). Nuclear factor kappa B in autism spectrum disorder: A systematic review. Pharmacol. Res..

[66] Strenn N., Hovey D., Jonsson L., Anckarster H., Anckarsäter H., Lundström S., Lichtenstein P., Ekman A. (2019). Associations between autistic-like traits and polymorphisms in NFKBIL1. Acta Neuropsychiatr..

