# Supplementary material for: Whole Exome Sequencing Identifies Novel De Novo Variants Interacting with Six Gene Networks in Autism Spectrum Disorder
Source: Genes (Basel). 2020 Dec 22;12(1):1. doi: 10.3390/genes12010001 (PMC7822011; doi:10.3390/genes12010001)
Supplement: Supplementary file 1 [file genes-12-00001-s001.zip › Supplementary Figure 1.pdf]

AMIGO1

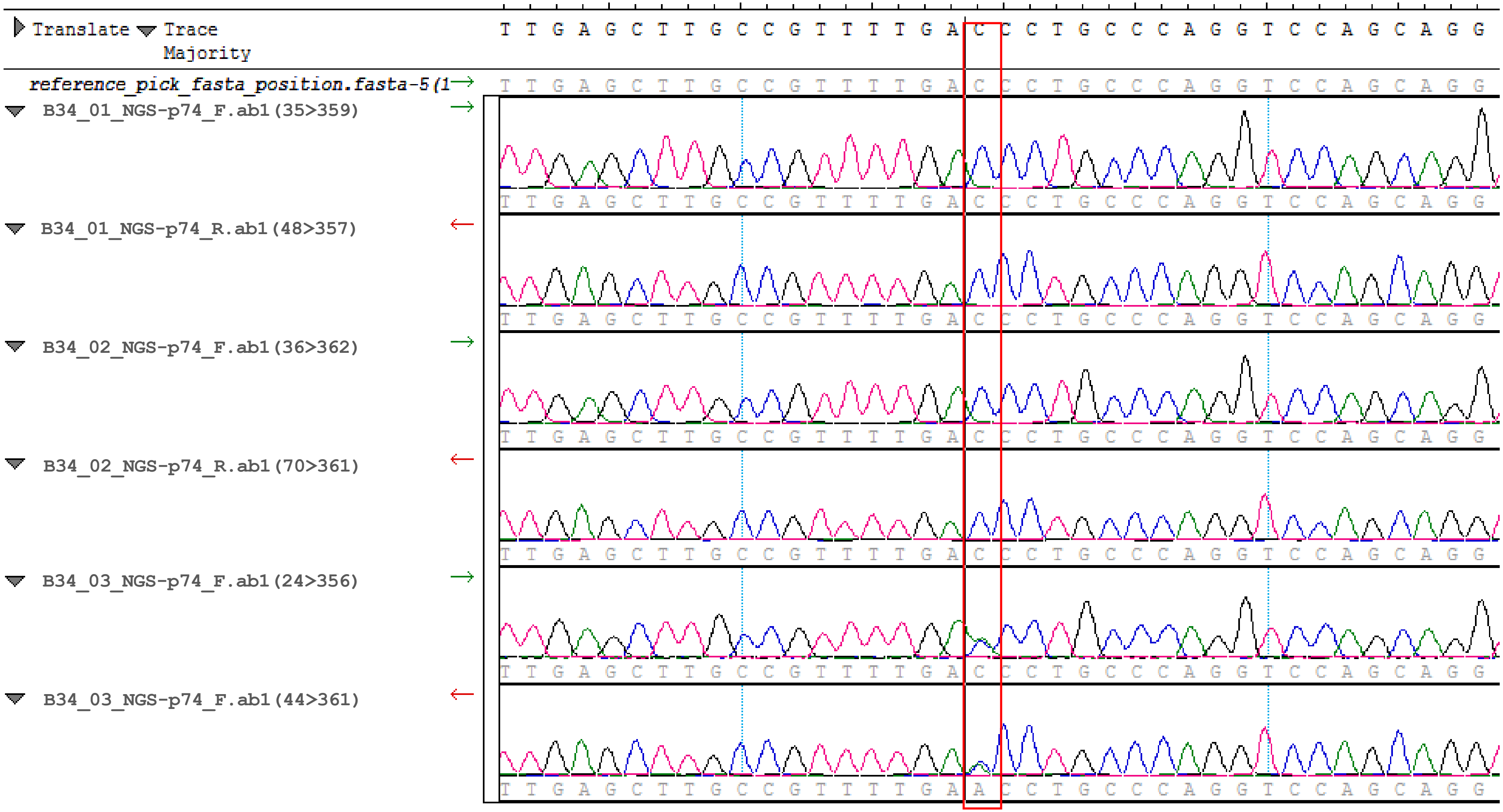

Translate
Trace
Majority
T T G A G C T T G C C G T T T T G A C C C T G C C C A G G T C C A G C A G G
reference_pick_fasta_position.fasta-5(1
B34_01_NGS-p74_F.ab1(35>359)
B34_01_NGS-p74_R.ab1(48>357)
B34_02_NGS-p74_F.ab1(36>362)
B34_02_NGS-p74_R.ab1(70>361)
B34_03_NGS-p74_F.ab1(24>356)
B34_03_NGS-p74_F.ab1(44>361)

HAX1

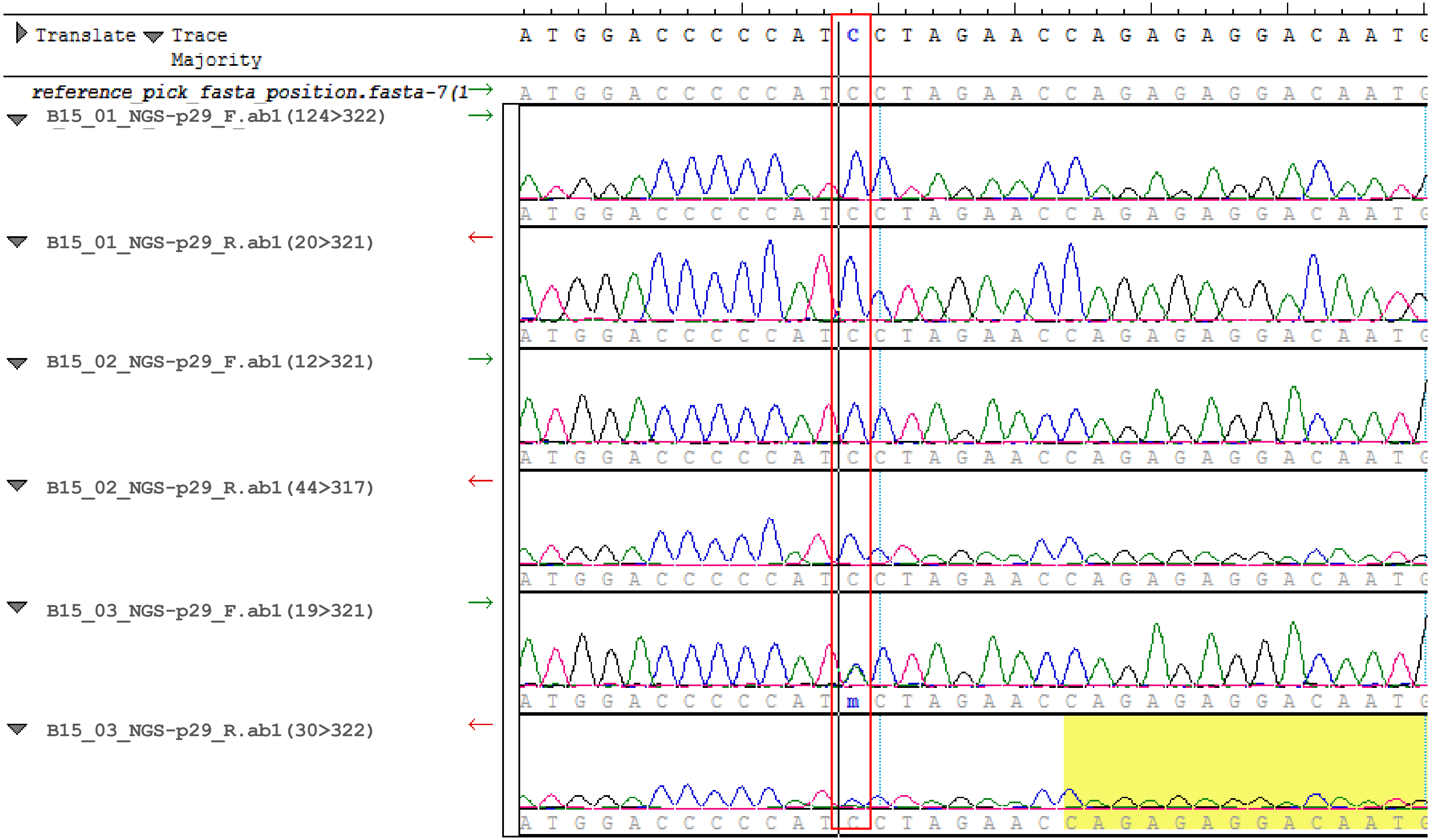
Translate
Trace
Majority
A T G G A C C C C C A T C C T A G A A C C A G A G A G G A C A A T G
reference_pick_fasta_position.fasta-7(1
B15_01_NGS-p29_F.ab1(124>322)
B15_01_NGS-p29_R.ab1(20>321)
B15_02_NGS-p29_F.ab1(12>321)
B15_02_NGS-p29_R.ab1(44>317)
B15_03_NGS-p29_F.ab1(19>321)
B15_03_NGS-p29_R.ab1(30>322)
A T G G A C C C C C A T m C T A G A A C C A G A G A G G A C A A T G

IWS1

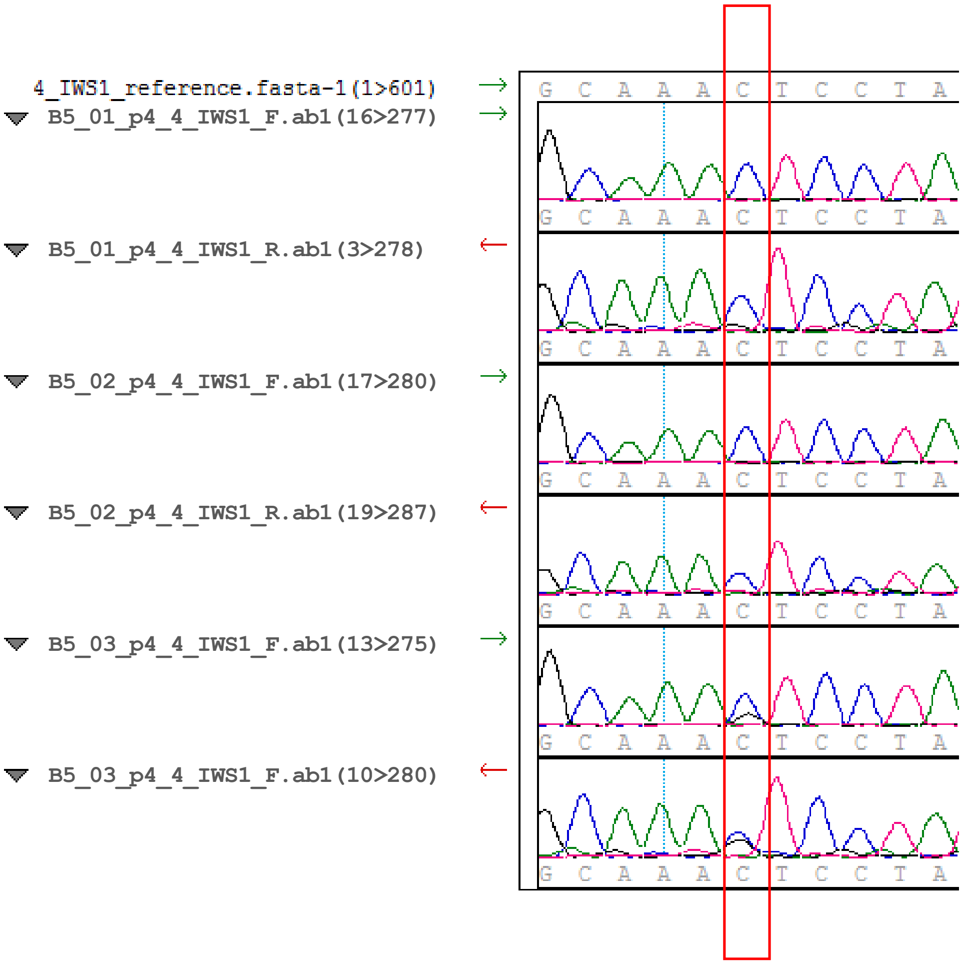

4_IWS1_reference.fasta-1(1>601)
B5_01_p4_4_IWS1_F.ab1(16>277)
B5_01_p4_4_IWS1_R.ab1(3>278)
B5_02_p4_4_IWS1_F.ab1(17>280)
B5_02_p4_4_IWS1_R.ab1(19>287)
B5_03_p4_4_IWS1_F.ab1(13>275)
B5_03_p4_4_IWS1_F.ab1(10>280)
G C A A A C T C C T A

TTC21A

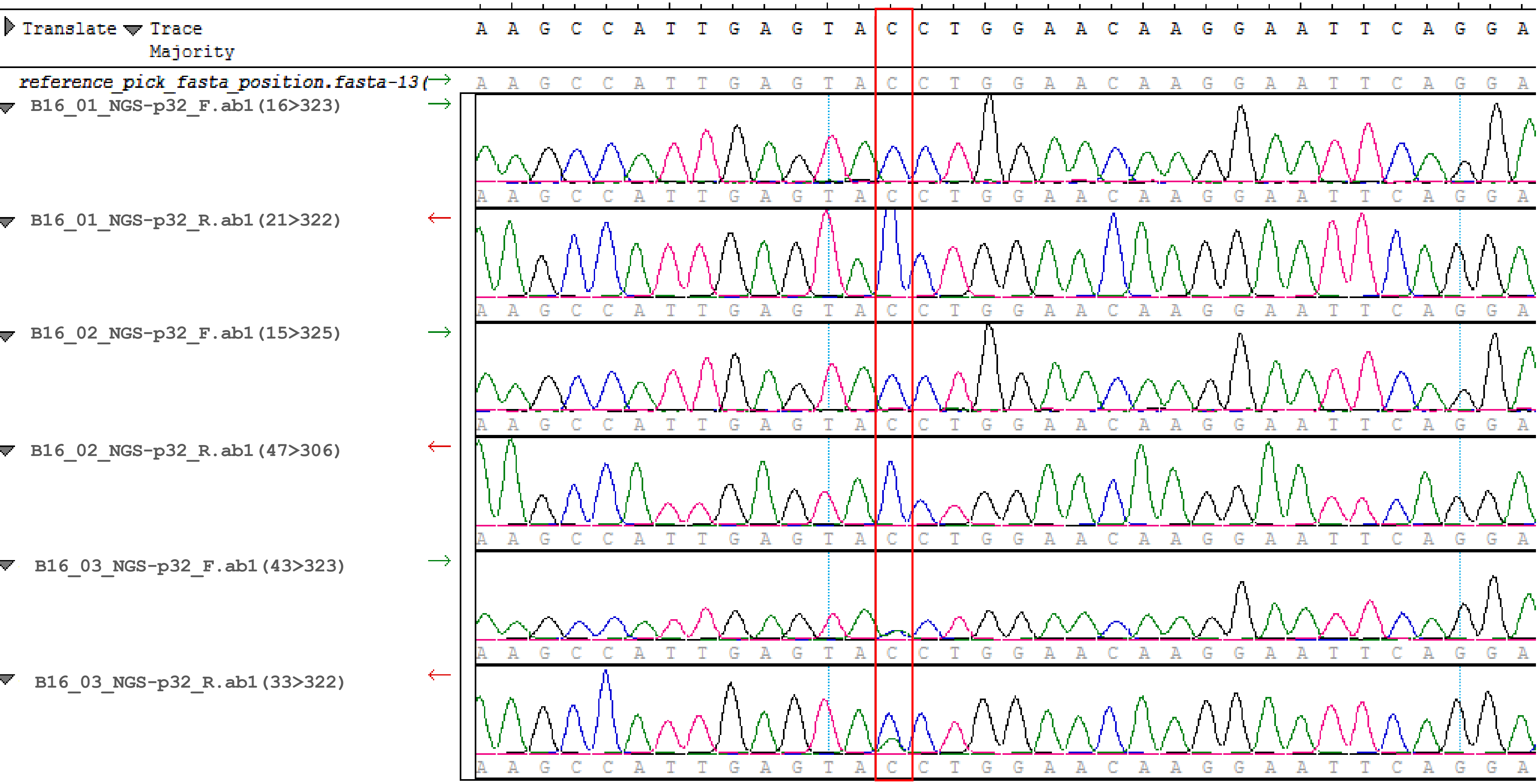
Translate Trace
Majority
A A G C C A T T G A G T A C C T G G A A C A A G G A A T T C A G G A
reference_pick_fasta_position.fasta-13(
B16_01_NGS-p32_F.ab1(16>323)
B16_01_NGS-p32_R.ab1(21>322)
B16_02_NGS-p32_F.ab1(15>325)
B16_02_NGS-p32_R.ab1(47>306)
B16_03_NGS-p32_F.ab1(43>323)
B16_03_NGS-p32_R.ab1(33>322)

LARS2

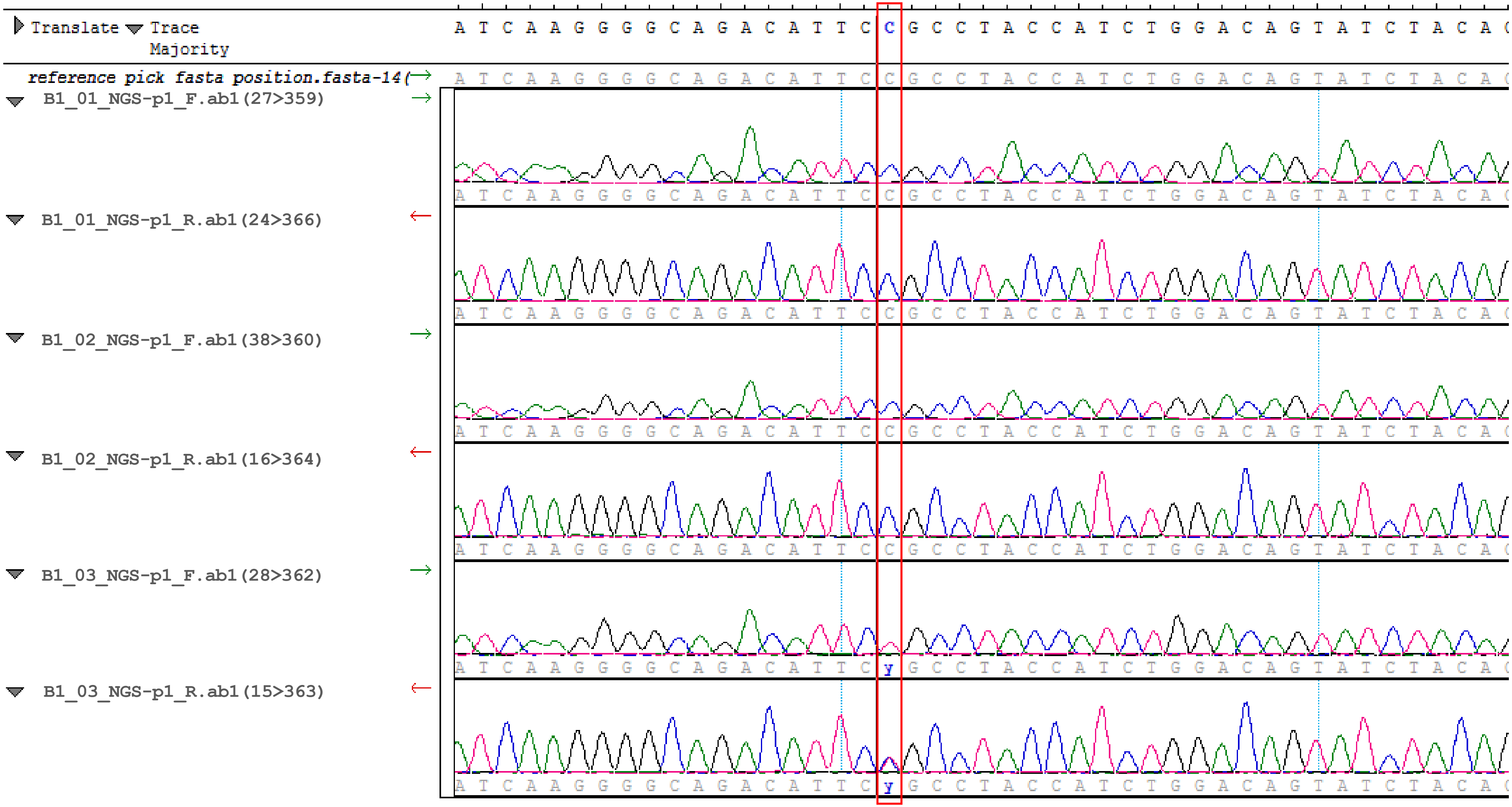

Translate
Trace
Majority
A T C A A G G G G C A G A C A T T C C G C C T A C C A T C T G G A C A G T A T C T A C A
reference pick fasta position.fasta-14
B1_01_NGS-p1_F.ab1(27>359)
B1_01_NGS-p1_R.ab1(24>366)
B1_02_NGS-p1_F.ab1(38>360)
B1_02_NGS-p1_R.ab1(16>364)
B1_03_NGS-p1_F.ab1(28>362)
B1_03_NGS-p1_R.ab1(15>363)

CELSR3

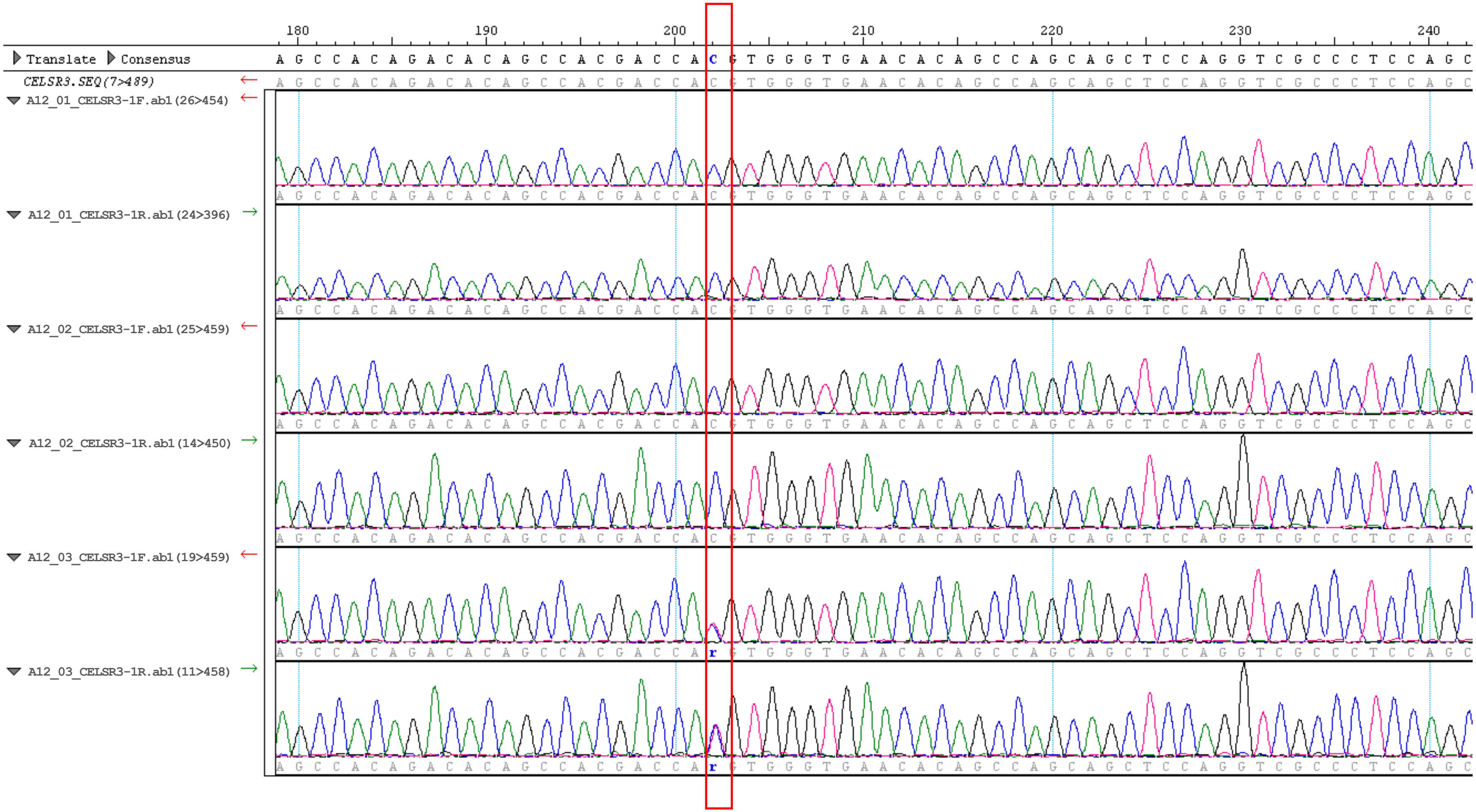

Translate
Consensus
CELSR3.SEQ(7>489)
A12_01_CELSR3-1F.ab1(26>454)
A12_01_CELSR3-1R.ab1(24>396)
A12_02_CELSR3-1F.ab1(25>459)
A12_02_CELSR3-1R.ab1(14>450)
A12_03_CELSR3-1F.ab1(19>459)
A12_03_CELSR3-1R.ab1(11>458)
180
190
200
210
220
230
240
A G C C A C A G A C A C A G C C A C G A C C A C G T G G G T G A A C A C A G C C A G C A G C T C C A G G T C G C C C T C C A G C

SLC26A1

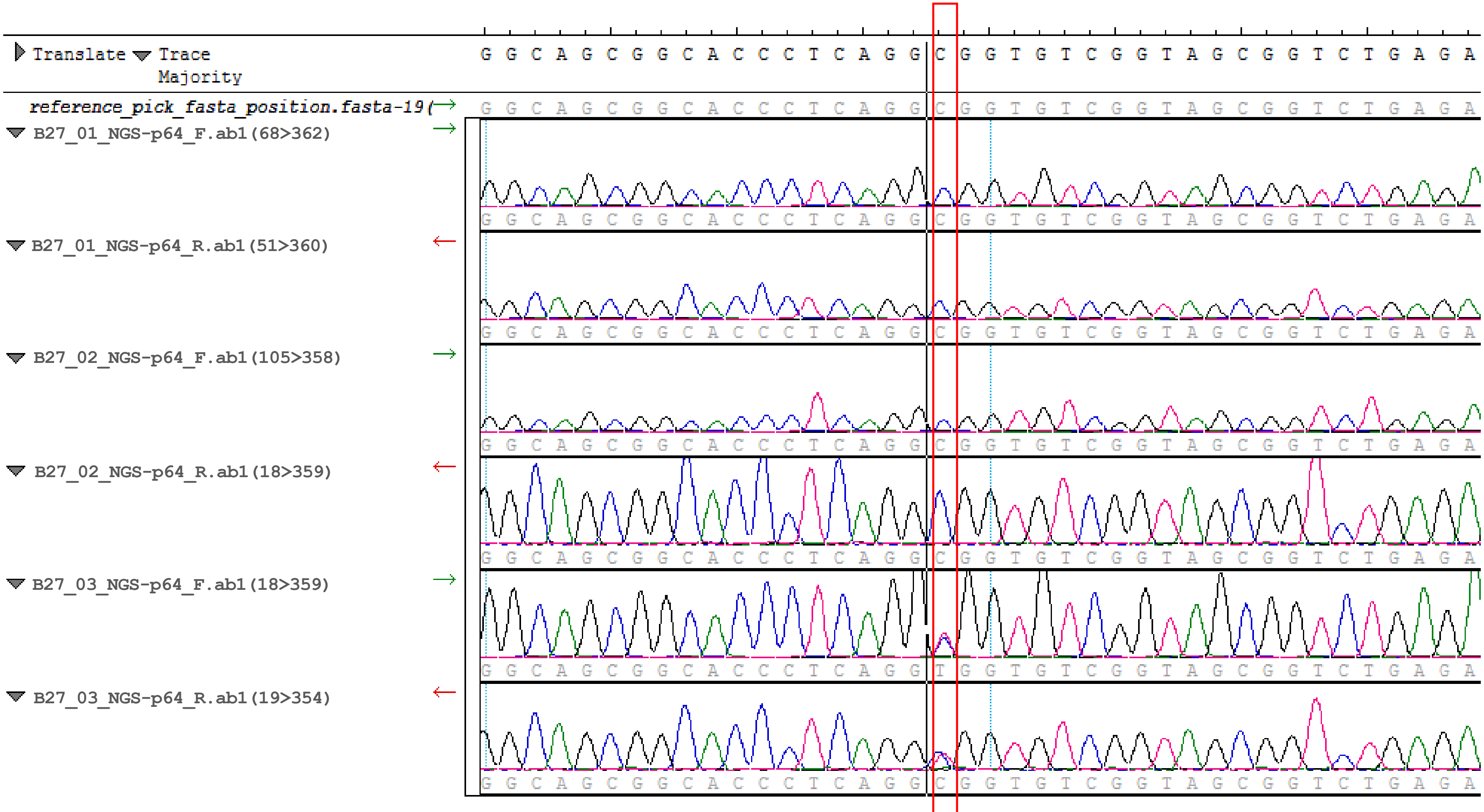

Translate
Trace
Majority
G G C A G C G G C A C C C T C A G G C G G T G T C G G T A G C G G T C T G A G A
reference_pick_fasta_position.fasta-19(
B27_01_NGS-p64_F.ab1(68>362)
B27_01_NGS-p64_R.ab1(51>360)
B27_02_NGS-p64_F.ab1(105>358)
B27_02_NGS-p64_R.ab1(18>359)
B27_03_NGS-p64_F.ab1(18>359)
B27_03_NGS-p64_R.ab1(19>354)

NFKB1

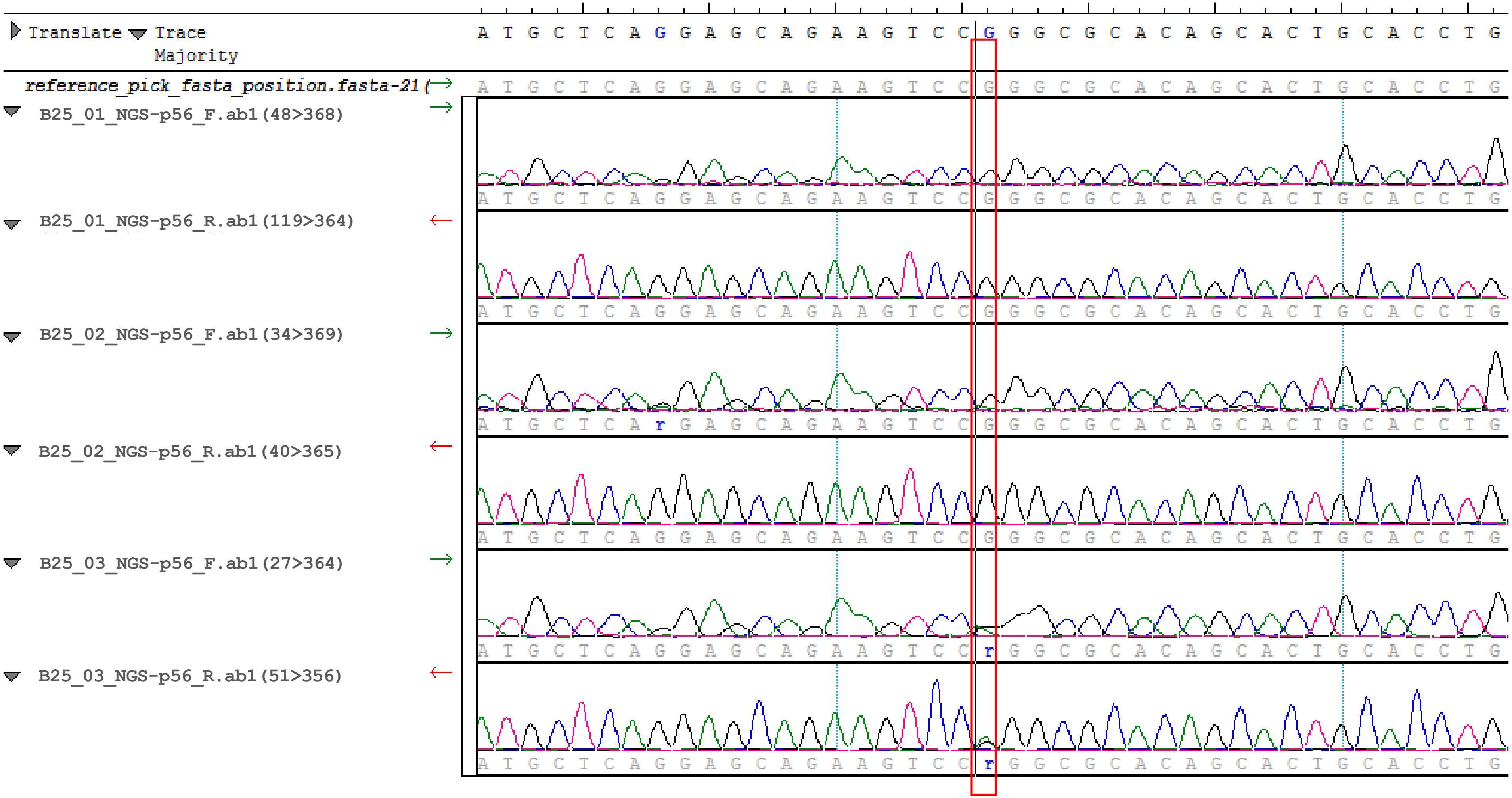

Translate Trace
Majority
ATGCTCAGGAGCAGAAGTCCGGGCGCACAGCACTGCACCTG
reference_pick_fasta_position.fasta-21
B25_01_NGS-p56_F.ab1(48>368)
B25_01_NGS-p56_R.ab1(119>364)
B25_02_NGS-p56_F.ab1(34>369)
B25_02_NGS-p56_R.ab1(40>365)
B25_03_NGS-p56_F.ab1(27>364)
B25_03_NGS-p56_R.ab1(51>356)

NEK1

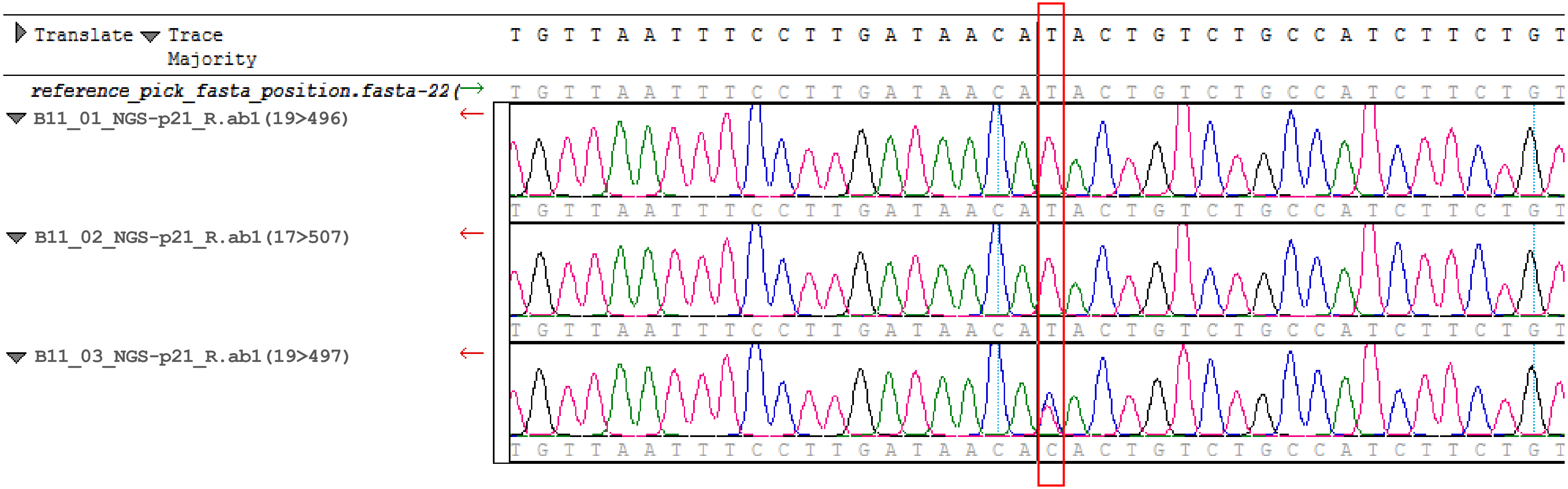

Translate
Trace
Majority
T G T T A A T T T C C T T G A T A A C A T A C T G T C T G C C A T C T T C T G T
reference_pick_fasta_position.fasta-22(
B11_01_NGS-p21_R.ab1(19>496)
B11_02_NGS-p21_R.ab1(17>507)
B11_03_NGS-p21_R.ab1(19>497)
T G T T A A T T T C C T T G A T A A C A C A C T G T C T G C C A T C T T C T G T

RBM27

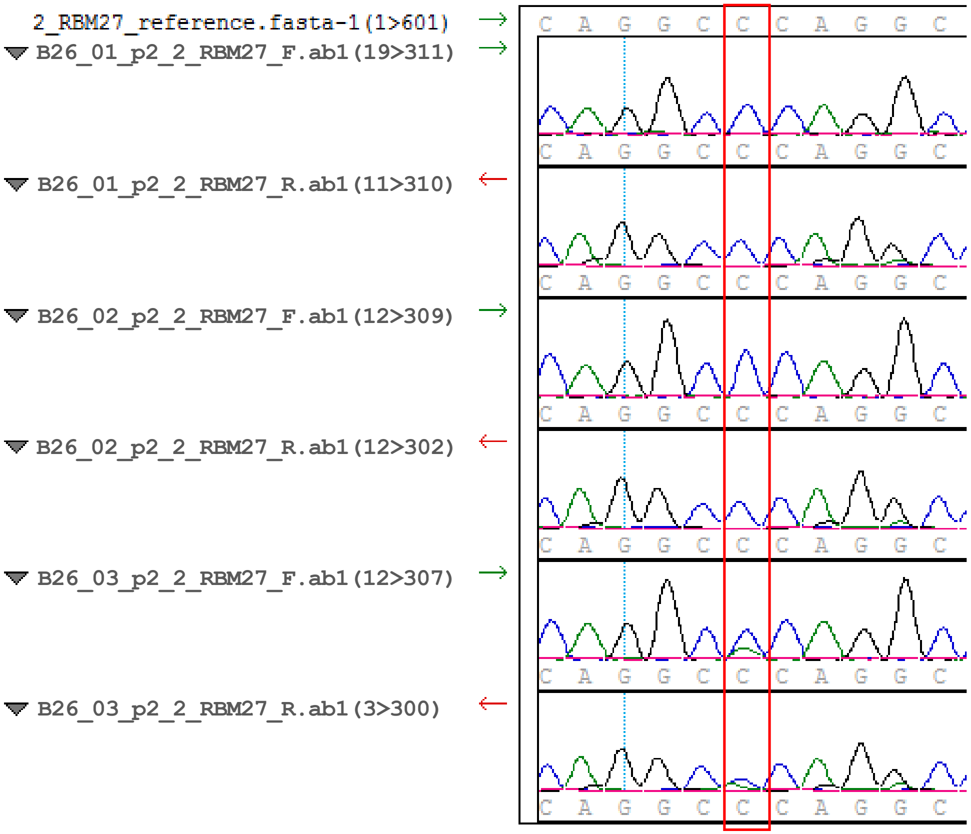

2_RBM27_reference.fasta-1(1>601)
B26_01_p2_2_RBM27_F.ab1(19>311)
B26_01_p2_2_RBM27_R.ab1(11>310)
B26_02_p2_2_RBM27_F.ab1(12>309)
B26_02_p2_2_RBM27_R.ab1(12>302)
B26_03_p2_2_RBM27_F.ab1(12>307)
B26_03_p2_2_RBM27_R.ab1(3>300)
C A G G C C C A G G C

RUFY1

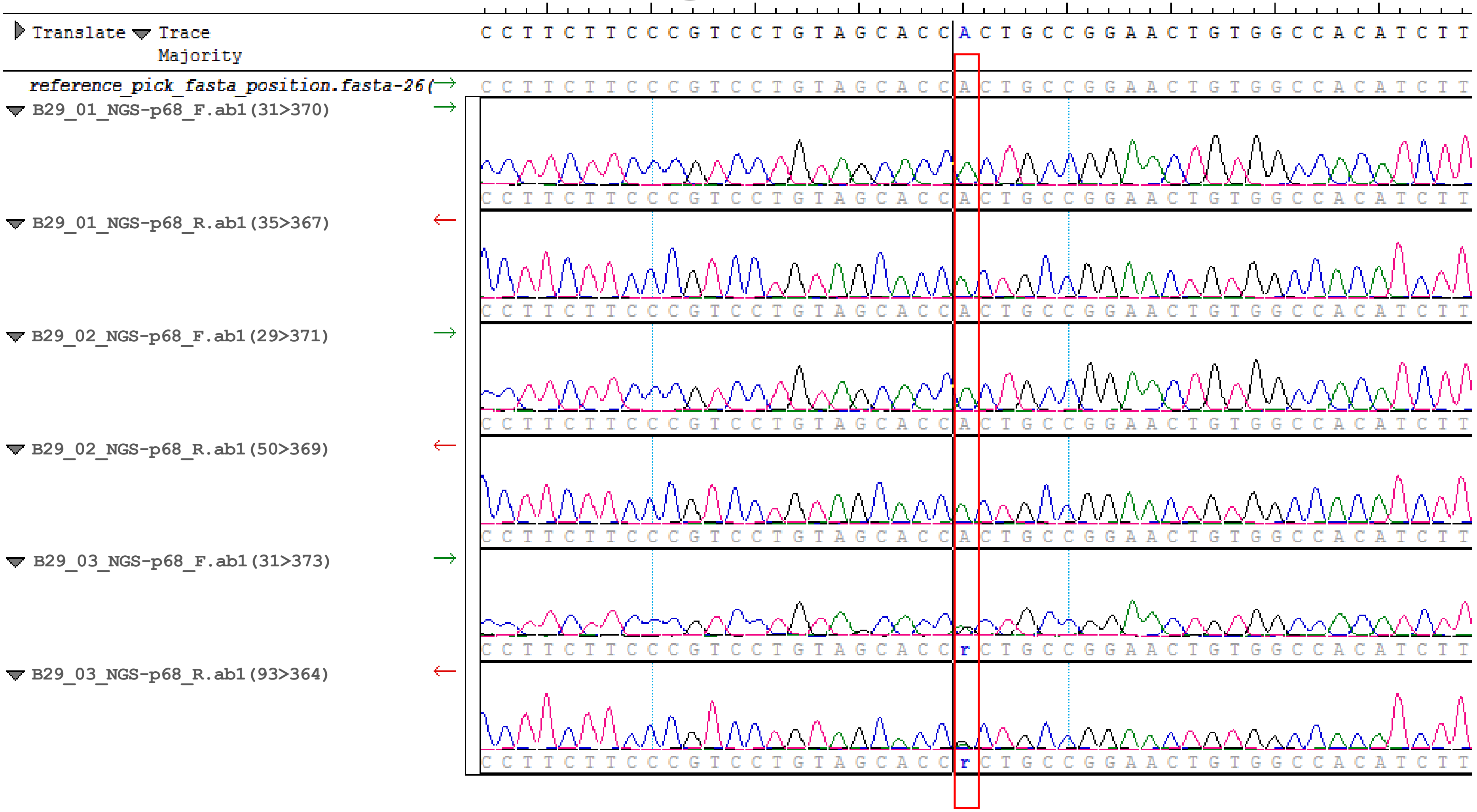

Translate
Trace
Majority
C C T T C T T C C C G T C C T G T A G C A C C A C T G C C G G A A C T G T G G C C A C A T C T T
reference_pick_fasta_position.fasta-26(
B29_01_NGS-p68_F.ab1(31>370)
B29_01_NGS-p68_R.ab1(35>367)
B29_02_NGS-p68_F.ab1(29>371)
B29_02_NGS-p68_R.ab1(50>369)
B29_03_NGS-p68_F.ab1(31>373)
B29_03_NGS-p68_R.ab1(93>364)
C C T T C T T C C C G T C C T G T A G C A C C r C T G C C G G A A C T G T G G C C A C A T C T T

ATXN1

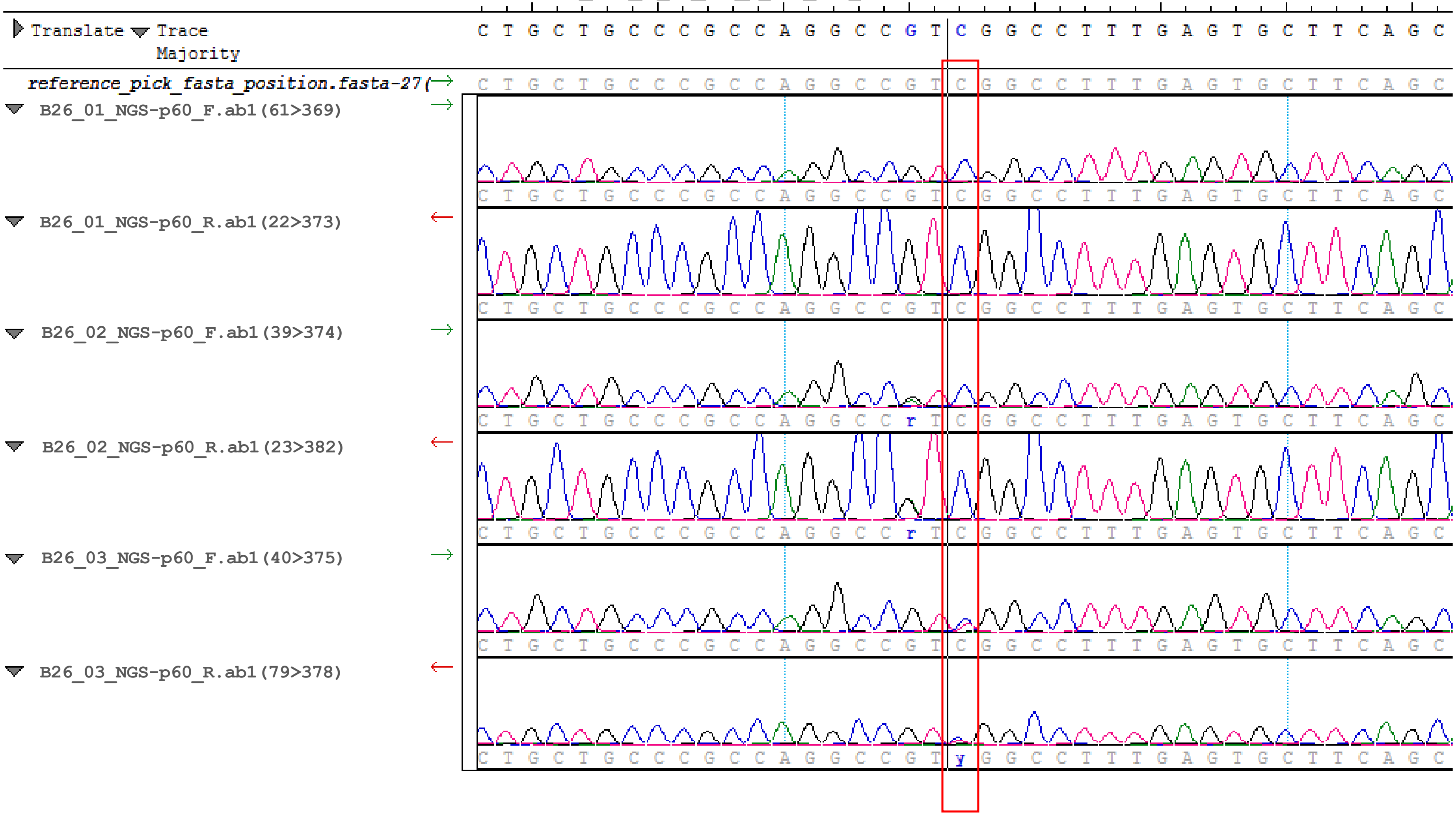
Translate
Trace
Majority
C T G C T G C C C G C C A G G C C G T C G G C C T T T G A G T G C T T C A G C
reference_pick_fasta_position.fasta-27
B26_01_NGS-p60_F.ab1(61>369)
B26_01_NGS-p60_R.ab1(22>373)
B26_02_NGS-p60_F.ab1(39>374)
B26_02_NGS-p60_R.ab1(23>382)
B26_03_NGS-p60_F.ab1(40>375)
B26_03_NGS-p60_R.ab1(79>378)

TCTE1

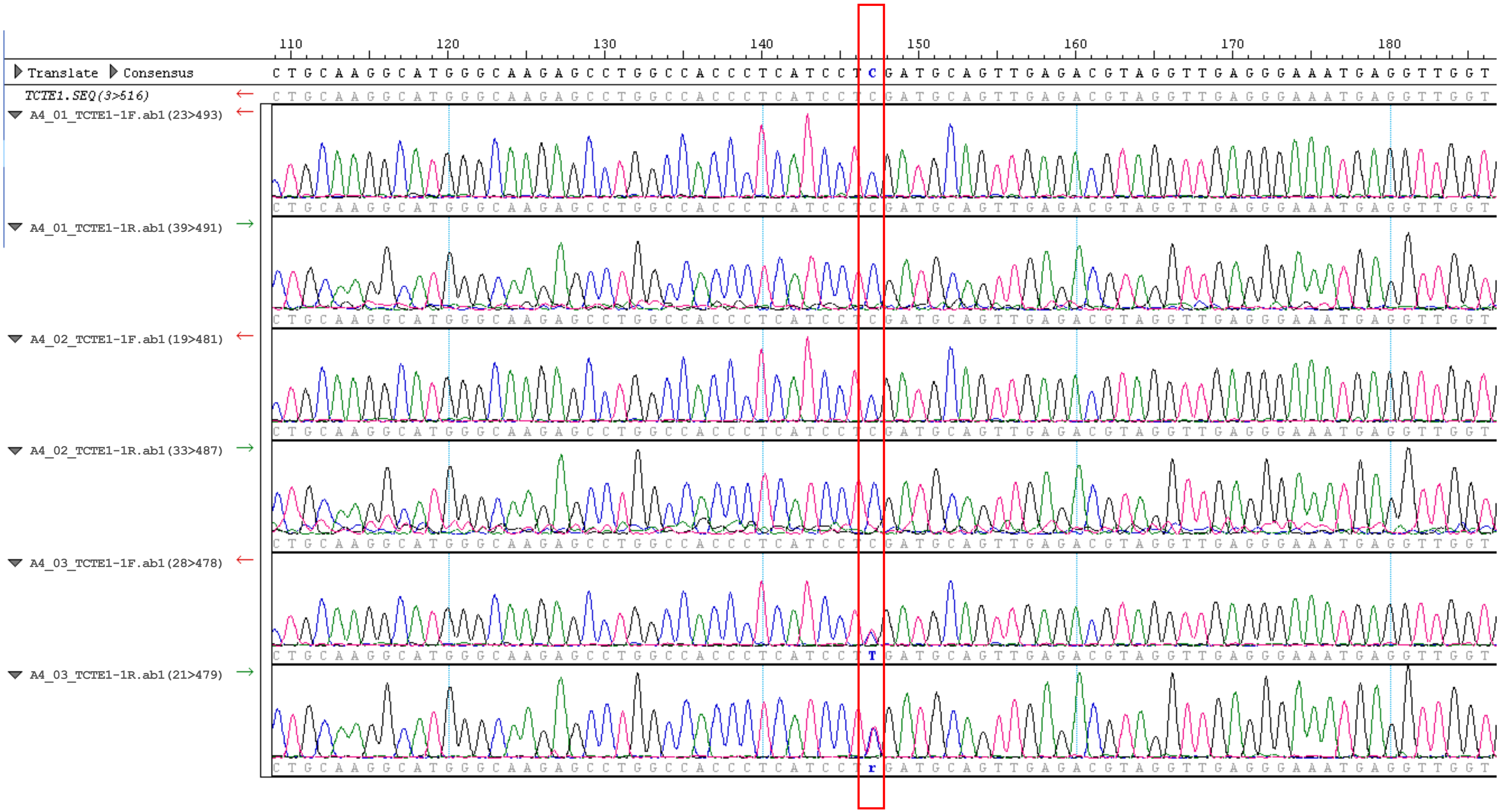
110
120
130
140
150
160
170
180
Translate
Consensus
TCTE1.SEQ(3>516)
A4_01_TCTE1-1F.ab1(23>493)
A4_01_TCTE1-1R.ab1(39>491)
A4_02_TCTE1-1F.ab1(19>481)
A4_02_TCTE1-1R.ab1(33>487)
A4_03_TCTE1-1F.ab1(28>478)
A4_03_TCTE1-1R.ab1(21>479)
C T G C A A G G C A T G G G C A A G A G C C T G G C C A C C C T C A T C C T C G A T G C A G T T G A G A C G T A G G T T G A G G G A A A T G A G G T T G G T

MTUS1

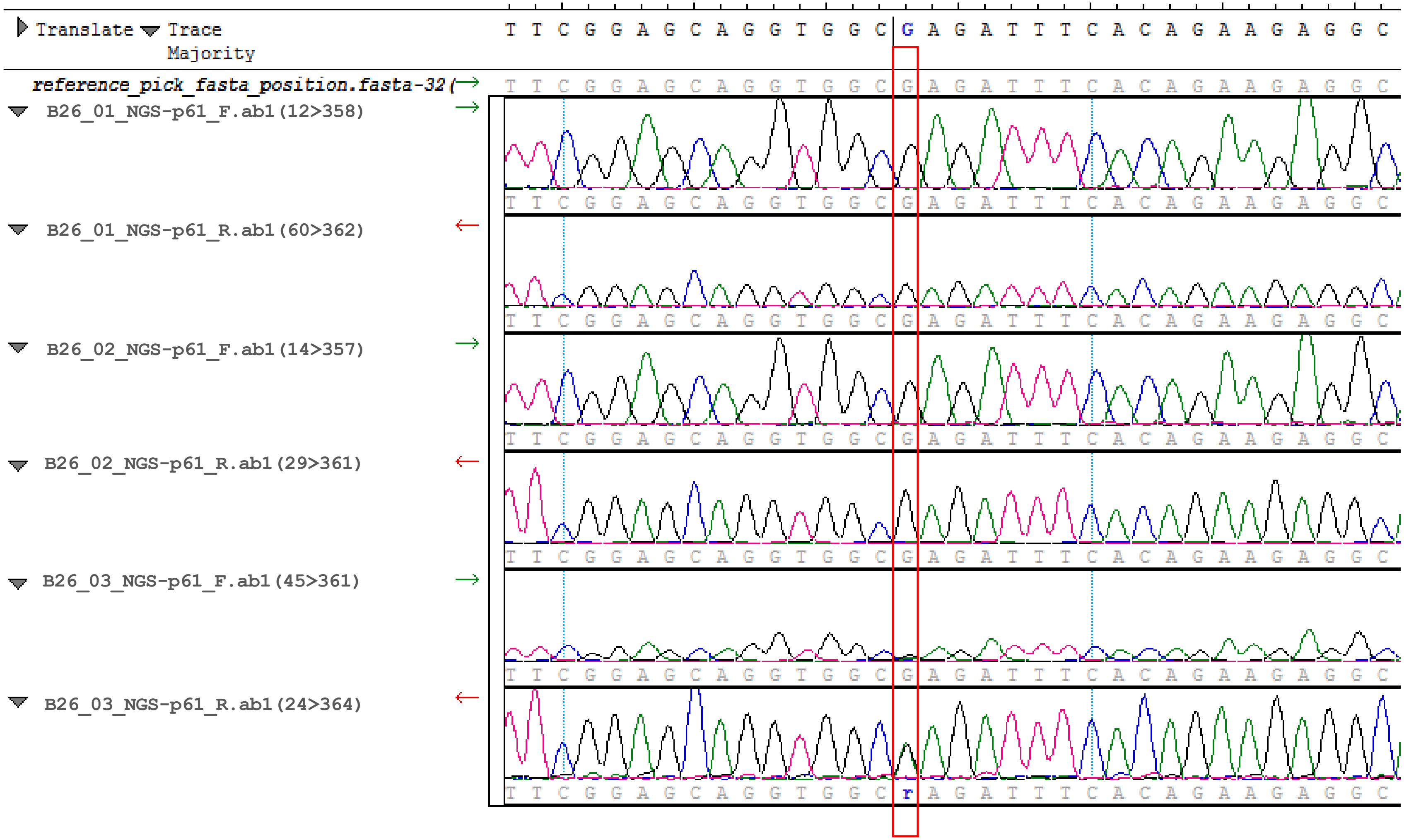

Translate
Trace
Majority
T T C G G A G C A G G T G G C G A G A T T T C A C A G A A G A G G C
reference_pick_fasta_position.fasta-32
B26_01_NGS-p61_F.ab1(12>358)
B26_01_NGS-p61_R.ab1(60>362)
B26_02_NGS-p61_F.ab1(14>357)
B26_02_NGS-p61_R.ab1(29>361)
B26_03_NGS-p61_F.ab1(45>361)
B26_03_NGS-p61_R.ab1(24>364)
T T C G G A G C A G G T G G C r A G A T T T C A C A G A A G A G G C

KCTD9

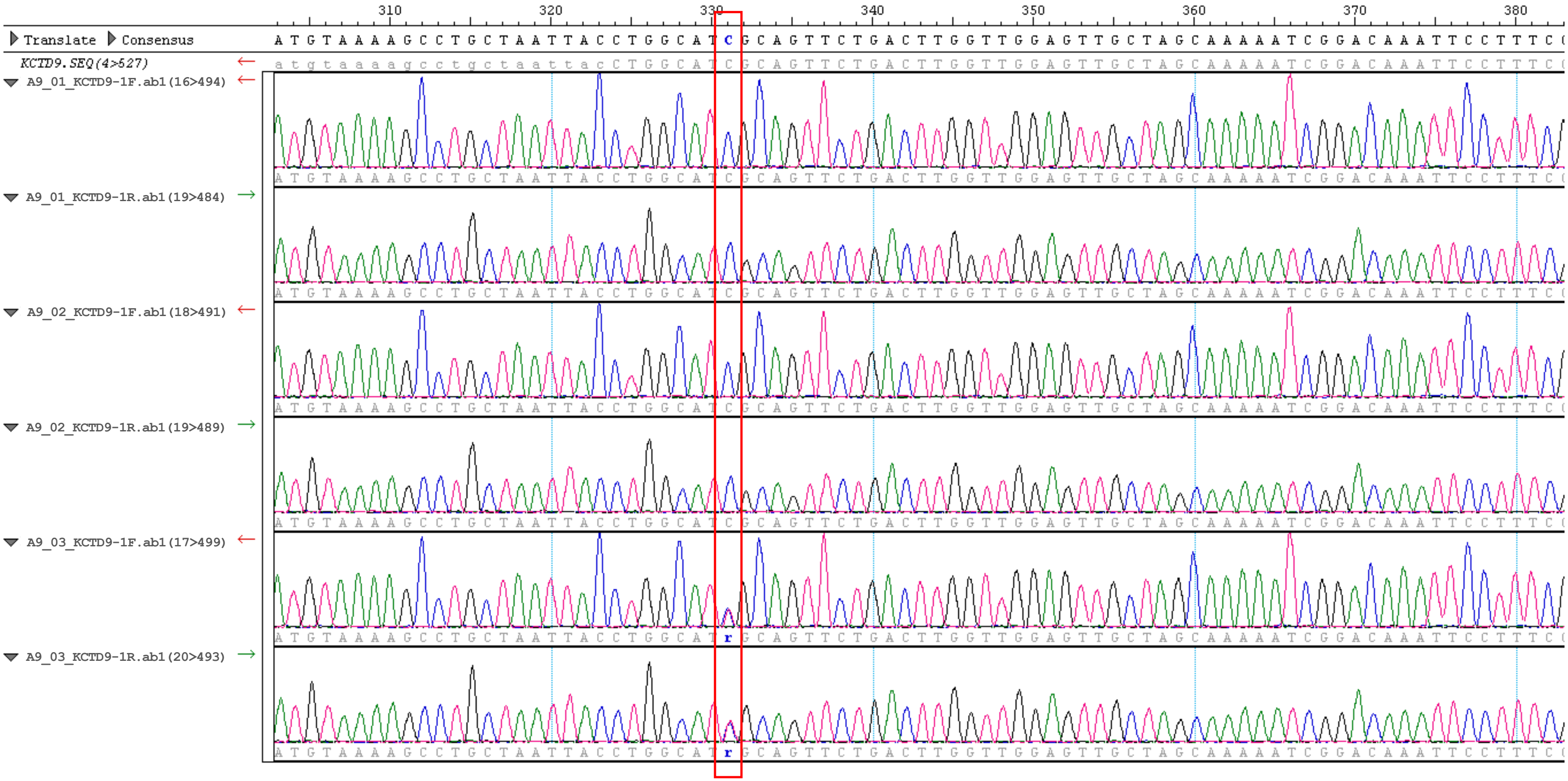

310
320
330
340
350
360
370
380
Translate
Consensus
A T G T A A A A G C C T G C T A A T T A C C T G G C A T C G C A G T T C T G A C T T G G T T G G A G T T G C T A G C A A A A A T C G G A C A A A T T C C T T T C
KCTD9.SEQ(4>527)
A9_01_KCTD9-1F.ab1(16>494)
A9_01_KCTD9-1R.ab1(19>484)
A9_02_KCTD9-1F.ab1(18>491)
A9_02_KCTD9-1R.ab1(19>489)
A9_03_KCTD9-1F.ab1(17>499)
A9_03_KCTD9-1R.ab1(20>493)

AKNA

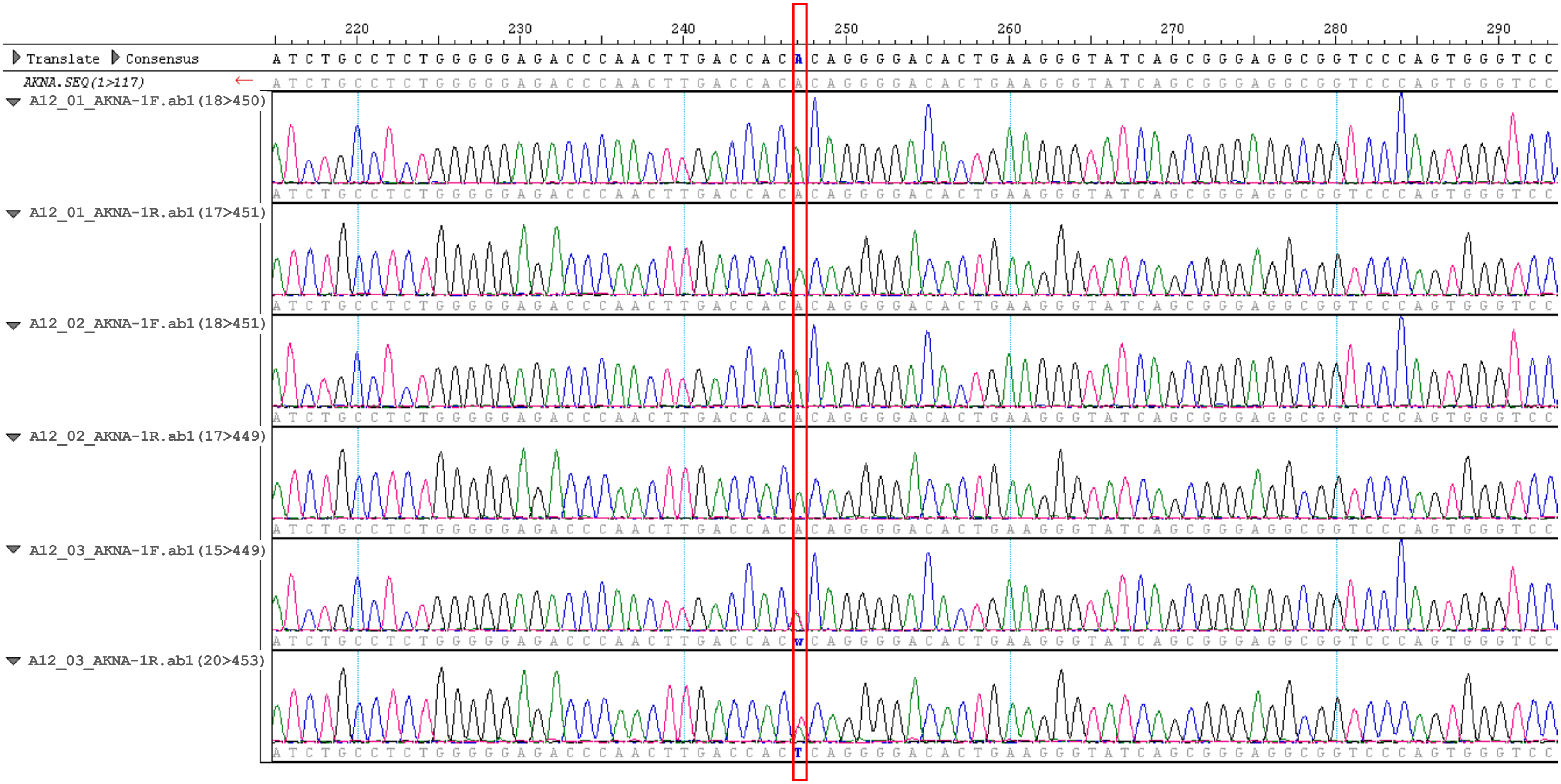

220 230 240 250 260 270 280 290
Translate
Consensus
ATCTGCCTCTGGGGGAGACCCAACTTGACCACACAGGGGACACTGAAGGGTATCAGCGGGAGGCGGTCCCAGTGGGTCC
AKNA.SEQ(1>117)
A12_01_AKNA-1F.ab1(18>450)
A12_01_AKNA-1R.ab1(17>451)
A12_02_AKNA-1F.ab1(18>451)
A12_02_AKNA-1R.ab1(17>449)
A12_03_AKNA-1F.ab1(15>449)
A12_03_AKNA-1R.ab1(20>453)

UBAC1

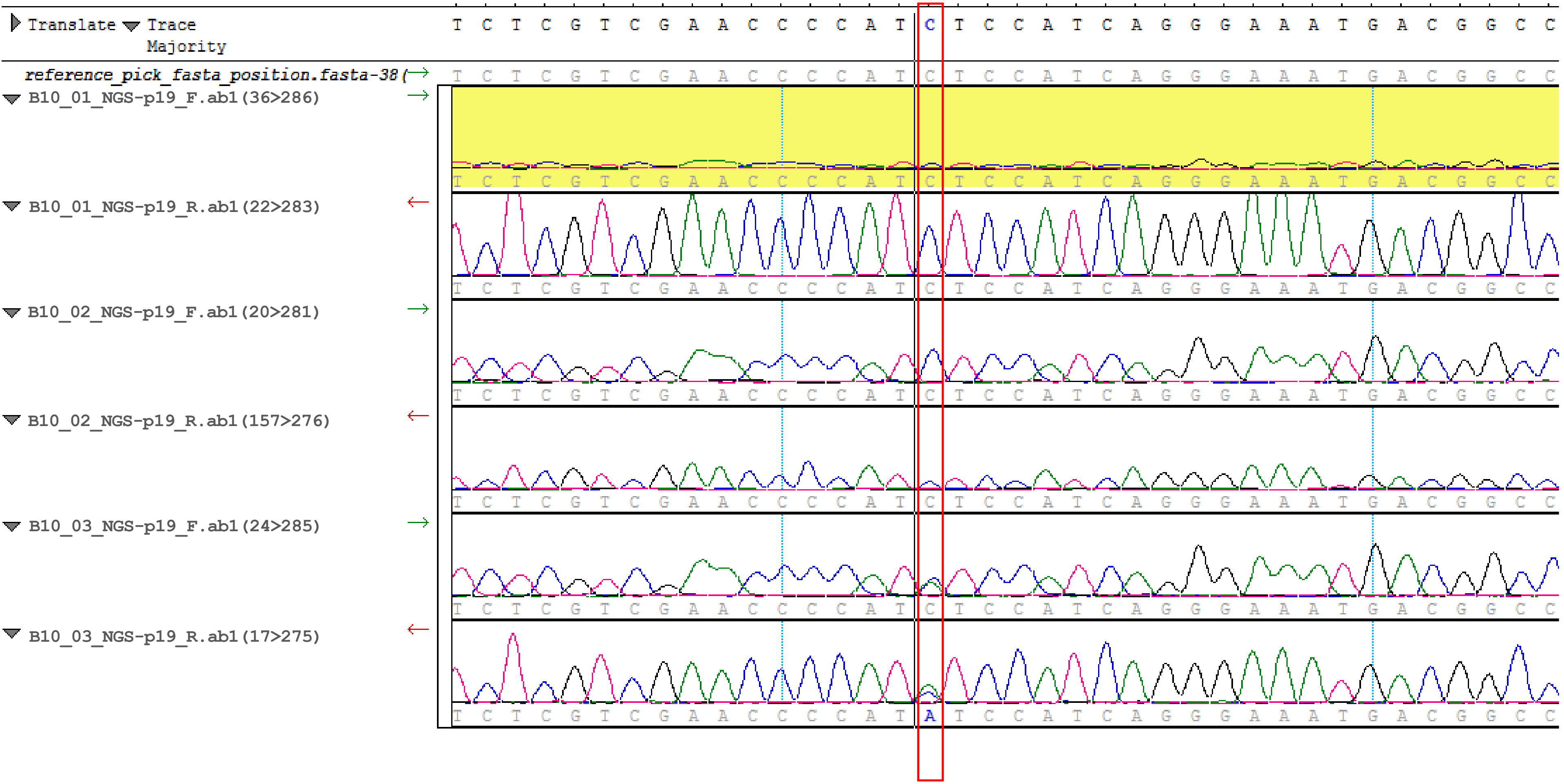
Translate
Trace
Majority
T C T C G T C G A A C C C C A T C T C C A T C A G G G A A A T G A C G G C C
reference_pick_fasta_position.fasta-38 (
B10_01_NGS-p19_F.ab1(36>286)
B10_01_NGS-p19_R.ab1(22>283)
B10_02_NGS-p19_F.ab1(20>281)
B10_02_NGS-p19_R.ab1(157>276)
B10_03_NGS-p19_F.ab1(24>285)
B10_03_NGS-p19_R.ab1(17>275)
T C T C G T C G A A C C C C A T A T C C A T C A G G G A A A T G A C G G C C

PITRM1-AS1

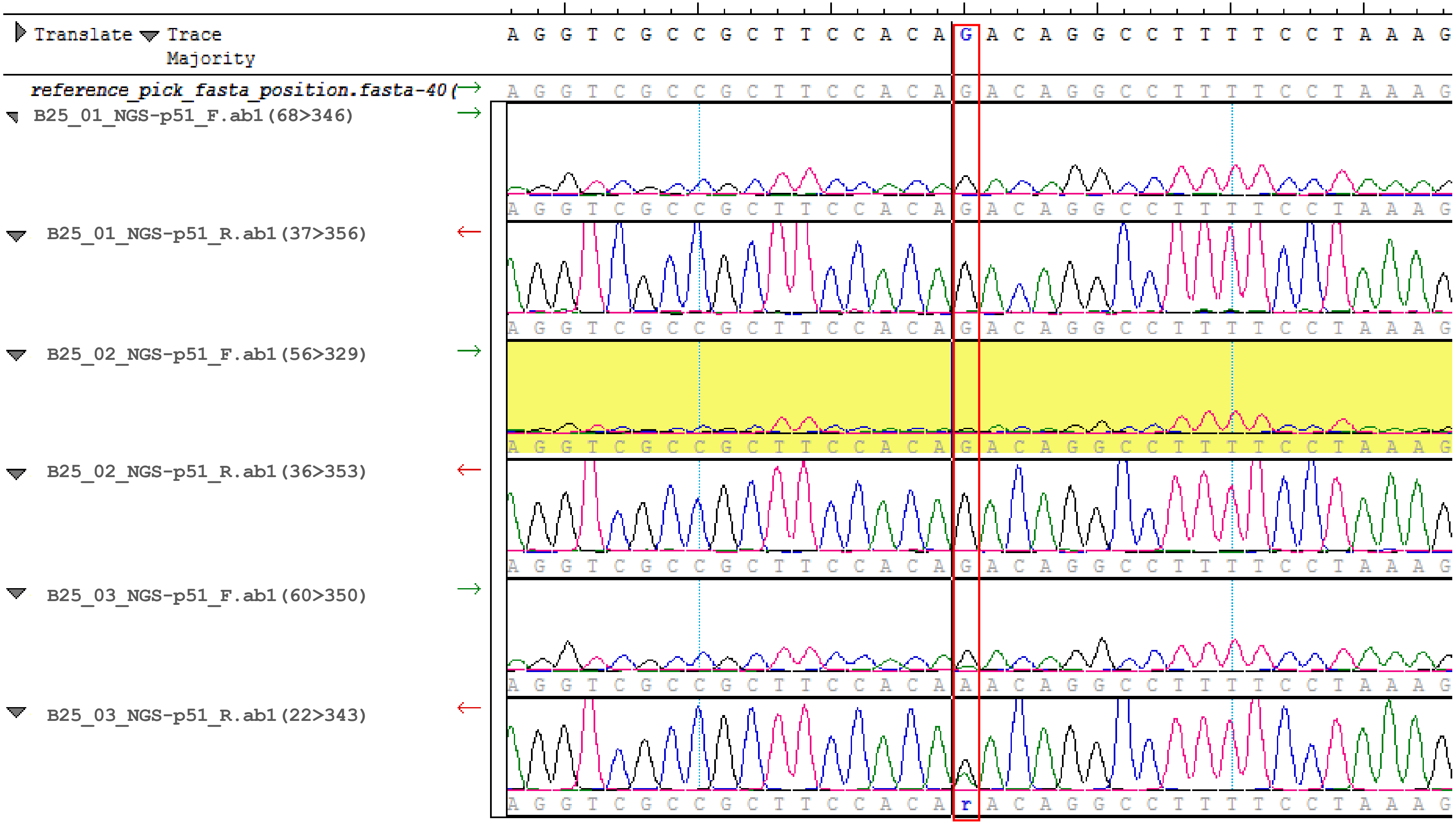
Translate
Trace
Majority
A G G T C G C C G C T T C C A C A G A C A G G C C T T T T C C T A A A G
reference_pick_fasta_position.fasta-40
B25_01_NGS-p51_F.ab1(68>346)
B25_01_NGS-p51_R.ab1(37>356)
B25_02_NGS-p51_F.ab1(56>329)
B25_02_NGS-p51_R.ab1(36>353)
B25_03_NGS-p51_F.ab1(60>350)
B25_03_NGS-p51_R.ab1(22>343)

SFMBT2

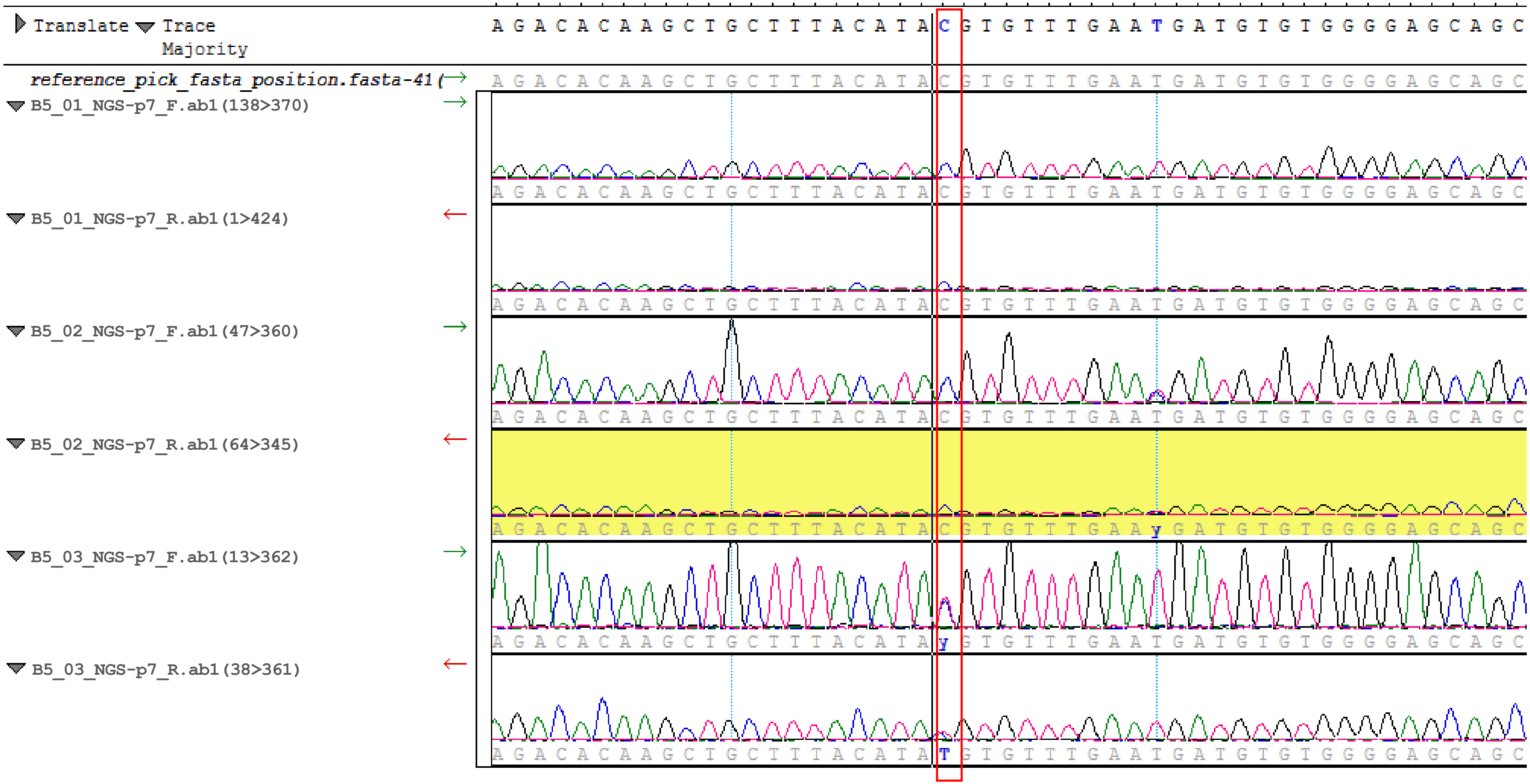

Translate
Trace
Majority
AGACACAAGCTGCTTTACATACGTGTTTGAATGATGTGTGGGGAGCAGC
reference_pick_fasta_position.fasta-41
B5_01_NGS-p7_F.ab1(138>370)
B5_01_NGS-p7_R.ab1(1>424)
B5_02_NGS-p7_F.ab1(47>360)
B5_02_NGS-p7_R.ab1(64>345)
B5_03_NGS-p7_F.ab1(13>362)
B5_03_NGS-p7_R.ab1(38>361)

RNH1

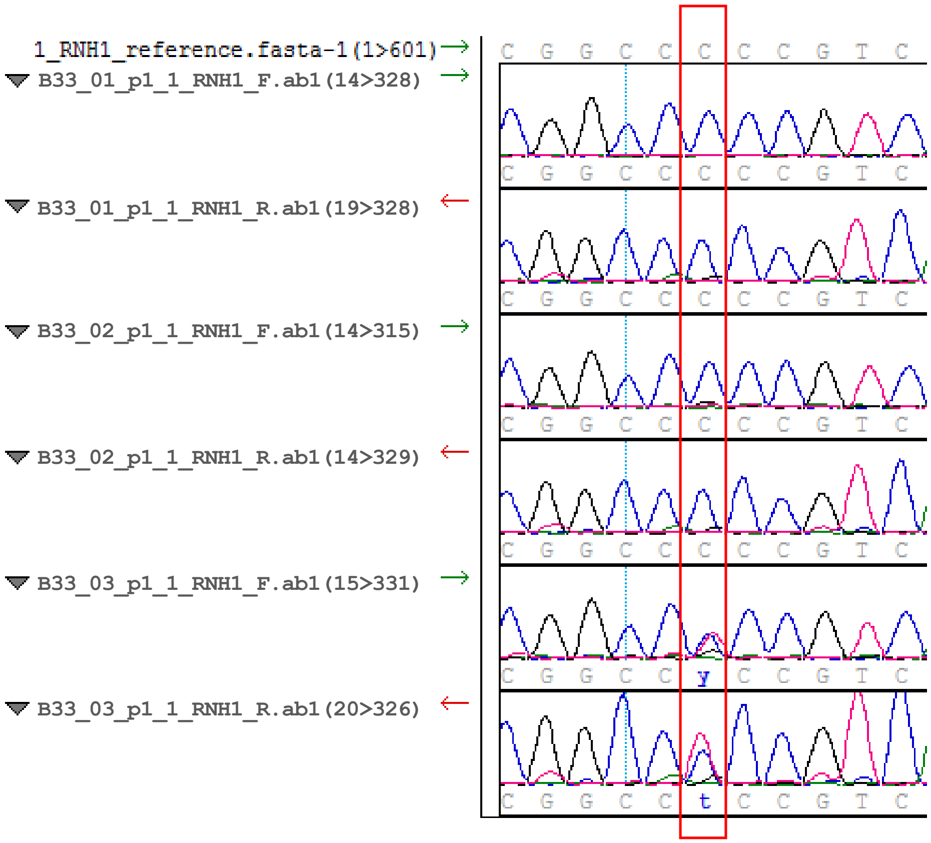

1_RNH1_reference.fasta-1(1>601)
B33_01_p1_1_RNH1_F.ab1(14>328)
B33_01_p1_1_RNH1_R.ab1(19>328)
B33_02_p1_1_RNH1_F.ab1(14>315)
B33_02_p1_1_RNH1_R.ab1(14>329)
B33_03_p1_1_RNH1_F.ab1(15>331)
B33_03_p1_1_RNH1_R.ab1(20>326)
C G G C C C C C G T C
C G G C C y C C G T C
C G G C C t C C G T C

DTX1

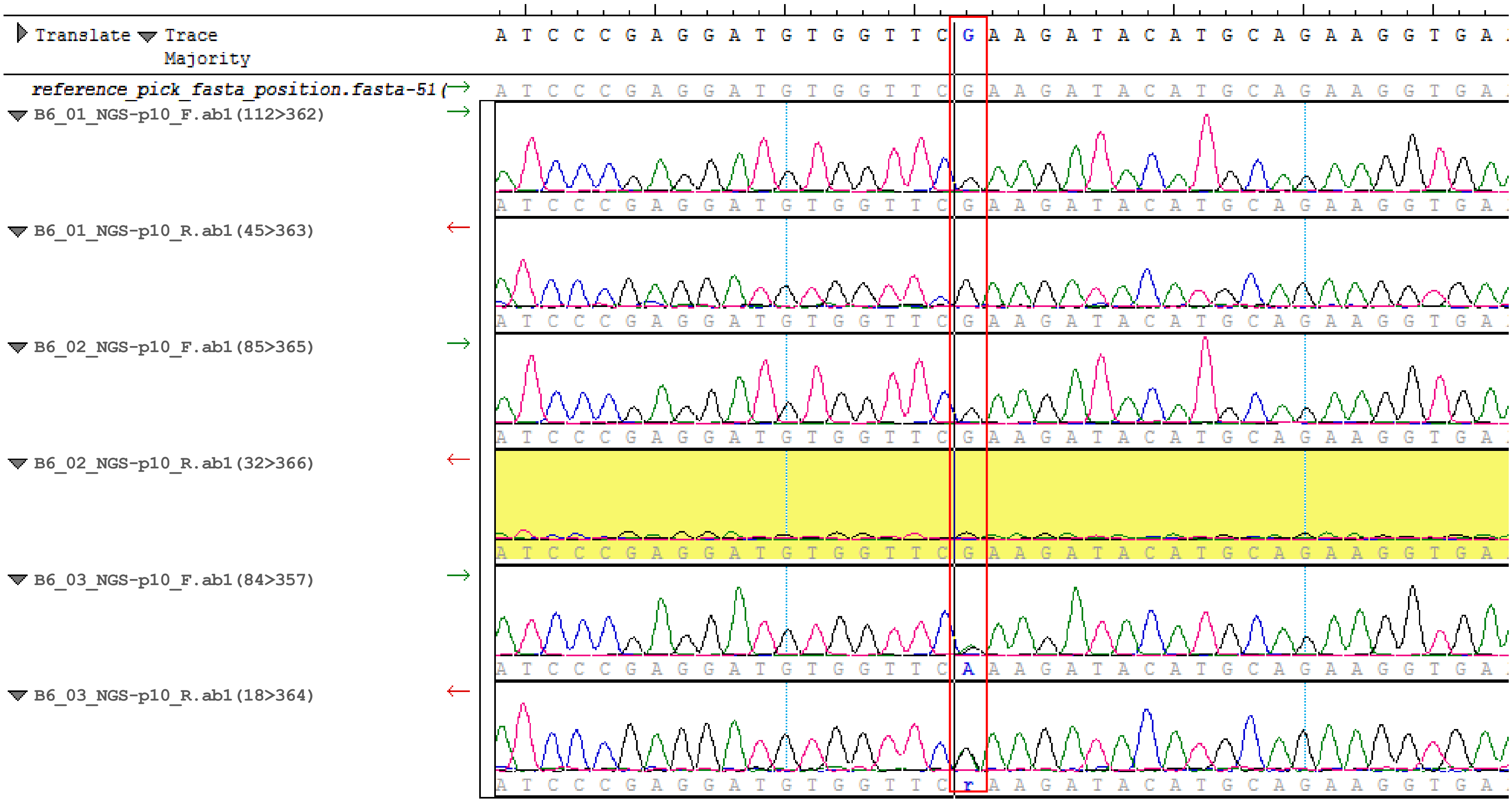

Translate Trace
Majority
A T C C C G A G G A T G T G G T T C G A A G A T A C A T G C A G A A G G T G A
reference_pick_fasta_position.fasta-51
B6_01_NGS-p10_F.ab1(112>362)
B6_01_NGS-p10_R.ab1(45>363)
B6_02_NGS-p10_F.ab1(85>365)
B6_02_NGS-p10_R.ab1(32>366)
B6_03_NGS-p10_F.ab1(84>357)
A T C C C G A G G A T G T G G T T C A A A G A T A C A T G C A G A A G G T G A
B6_03_NGS-p10_R.ab1(18>364)
A T C C C G A G G A T G T G G T T C r A A G A T A C A T G C A G A A G G T G A

RASGRP1

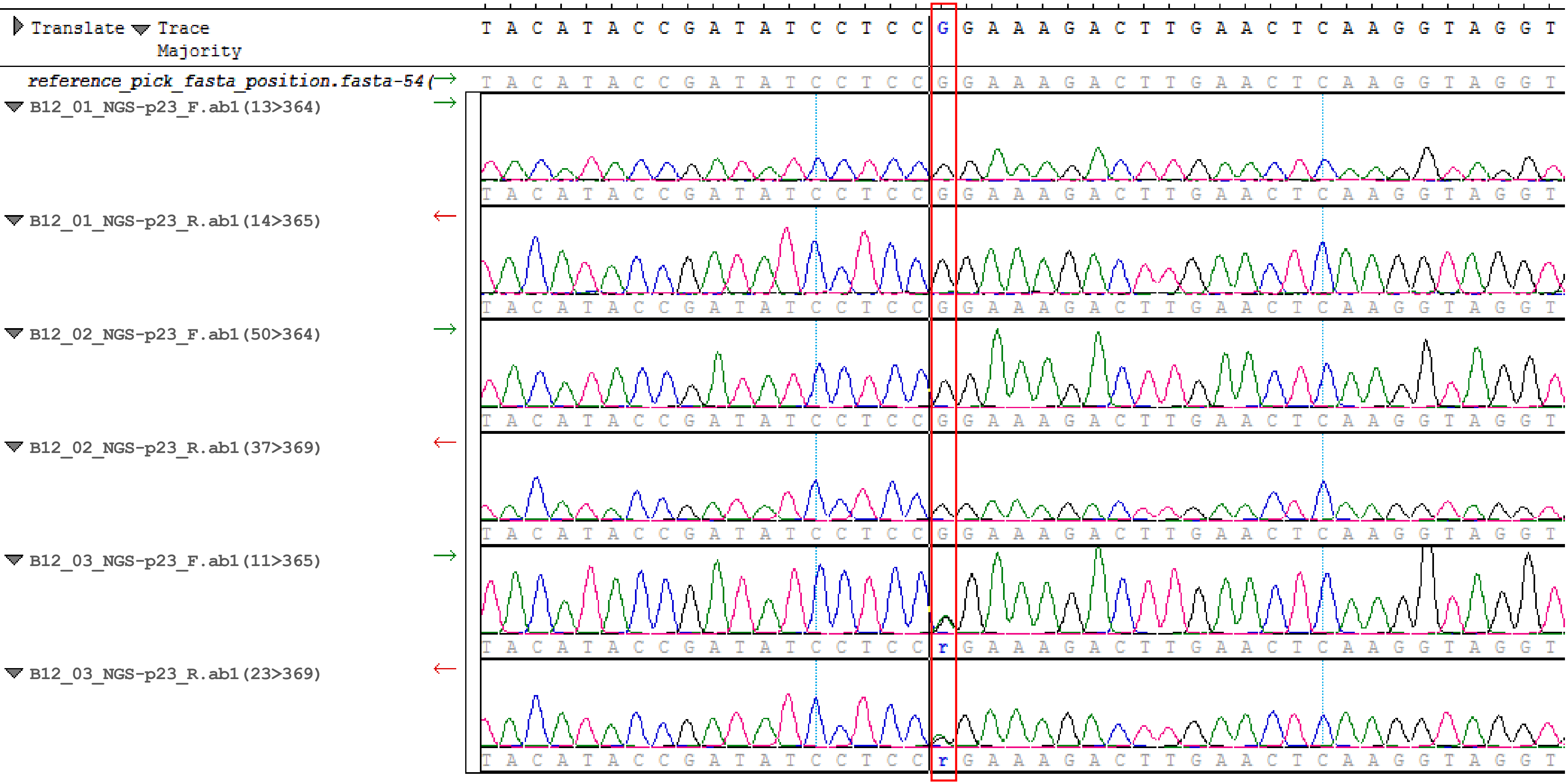

Translate
Trace
Majority
T A C A T A C C G A T A T C C T C C G G A A A G A C T T G A A C T C A A G G T A G G T
reference_pick_fasta_position.fasta-54(
B12_01_NGS-p23_F.ab1(13>364)
B12_01_NGS-p23_R.ab1(14>365)
B12_02_NGS-p23_F.ab1(50>364)
B12_02_NGS-p23_R.ab1(37>369)
B12_03_NGS-p23_F.ab1(11>365)
B12_03_NGS-p23_R.ab1(23>369)

SYNM

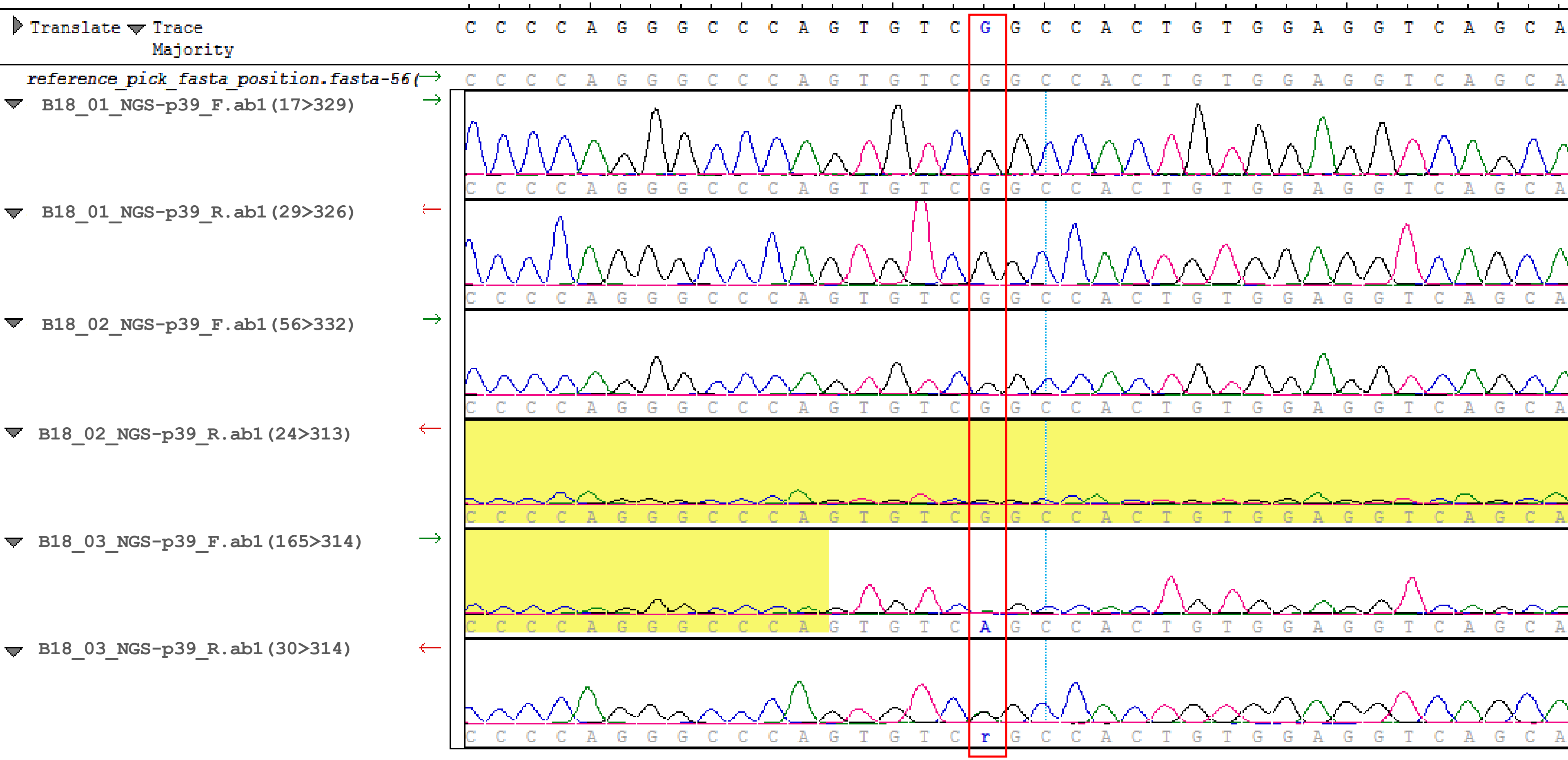
Translate
Trace
Majority
C C C C A G G G C C C A G T G T C G G C C A C T G T G G A G G T C A G C A
reference_pick_fasta_position.fasta-56
B18_01_NGS-p39_F.ab1(17>329)
B18_01_NGS-p39_R.ab1(29>326)
B18_02_NGS-p39_F.ab1(56>332)
B18_02_NGS-p39_R.ab1(24>313)
B18_03_NGS-p39_F.ab1(165>314)
B18_03_NGS-p39_R.ab1(30>314)

TMEM8A

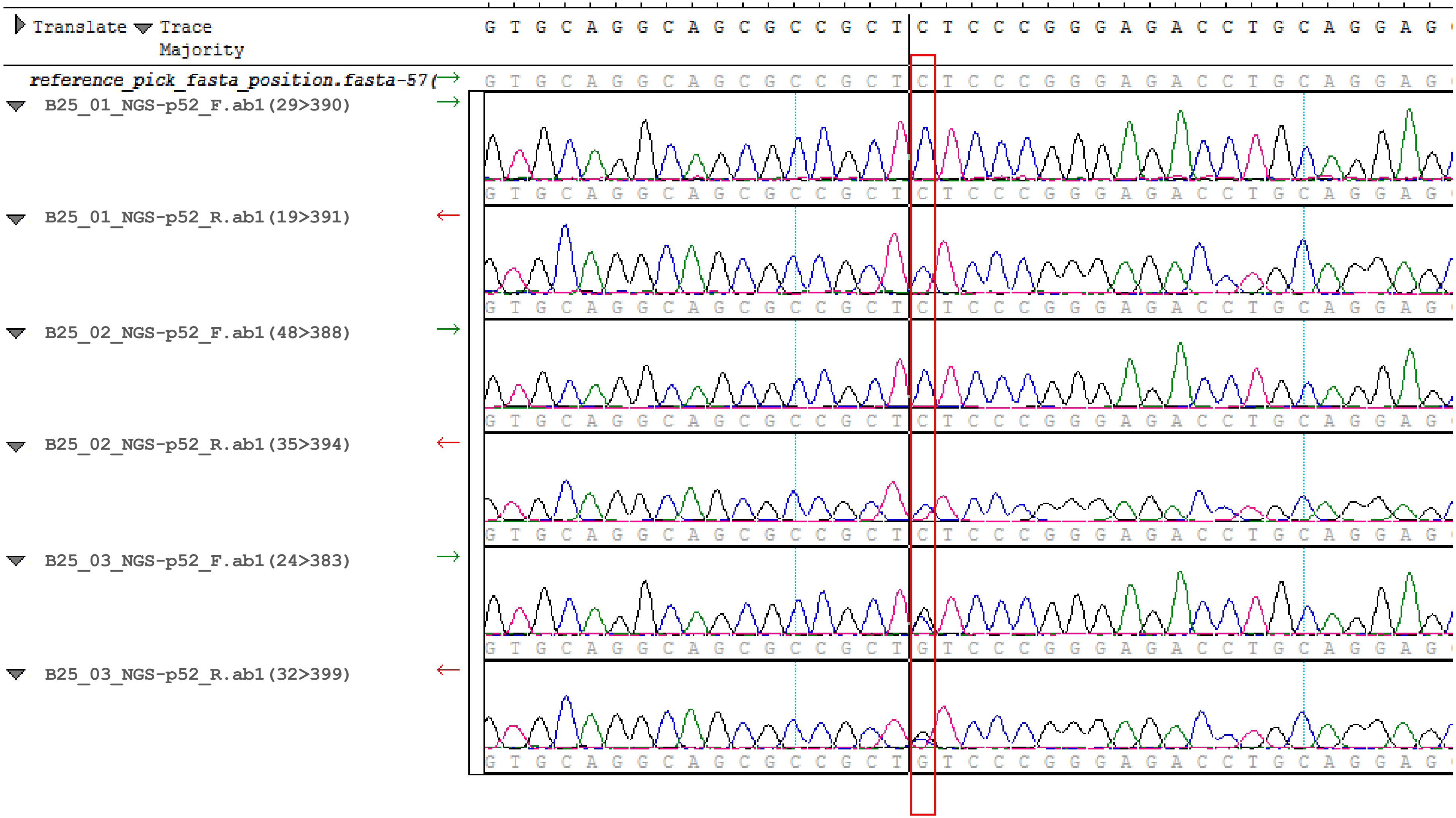

Translate
Trace
Majority
G T G C A G G C A G C G C C G C T C T C C C G G G A G A C C T G C A G G A G
reference_pick_fasta_position.fasta-57(
B25_01_NGS-p52_F.ab1(29>390)
B25_01_NGS-p52_R.ab1(19>391)
B25_02_NGS-p52_F.ab1(48>388)
B25_02_NGS-p52_R.ab1(35>394)
B25_03_NGS-p52_F.ab1(24>383)
B25_03_NGS-p52_R.ab1(32>399)

ADCY7

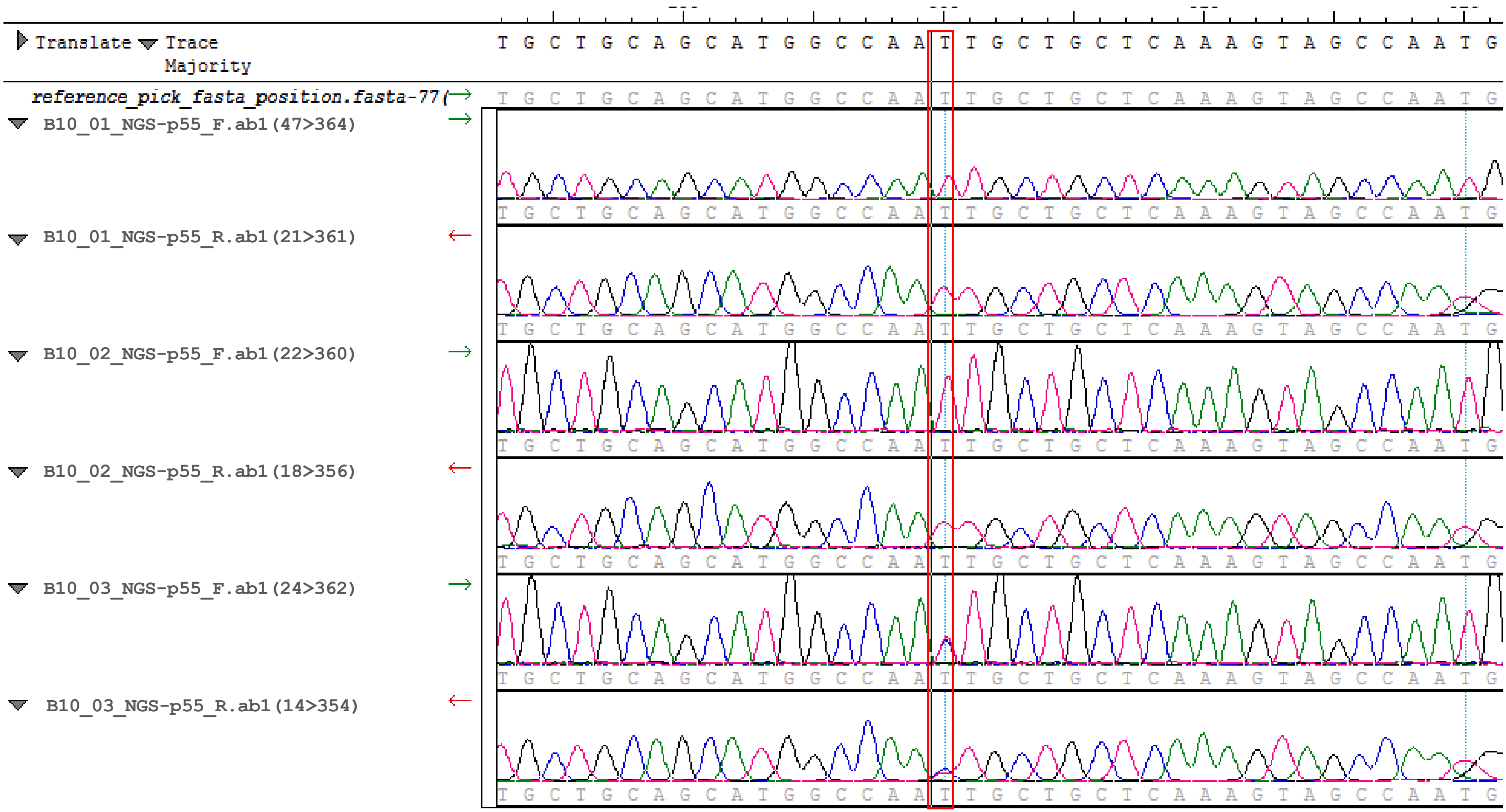

Translate
Trace
Majority
T G C T G C A G C A T G G C C A A T T G C T G C T C A A A G T A G C C A A T G
reference_pick_fasta_position.fasta-77(
B10_01_NGS-p55_F.ab1(47>364)
B10_01_NGS-p55_R.ab1(21>361)
B10_02_NGS-p55_F.ab1(22>360)
B10_02_NGS-p55_R.ab1(18>356)
B10_03_NGS-p55_F.ab1(24>362)
B10_03_NGS-p55_R.ab1(14>354)

PIEZO1

Comment
2_C2108-03 : heterozygosity (del GCCGTGACTCGGAAACGAGCGGCCA)
2_C2108-01 : chr16:88804927 : del C (other variant)

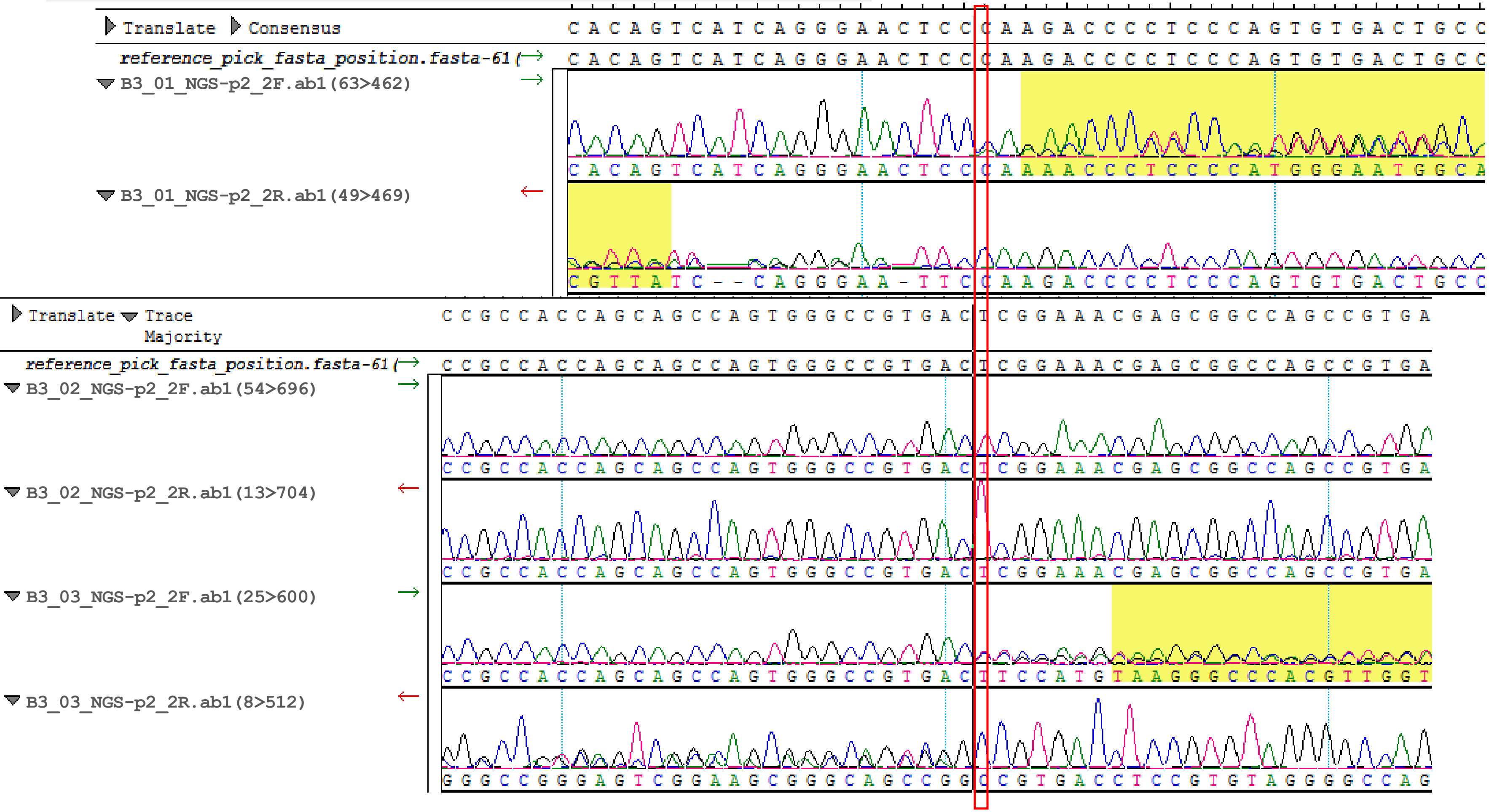

SNF8

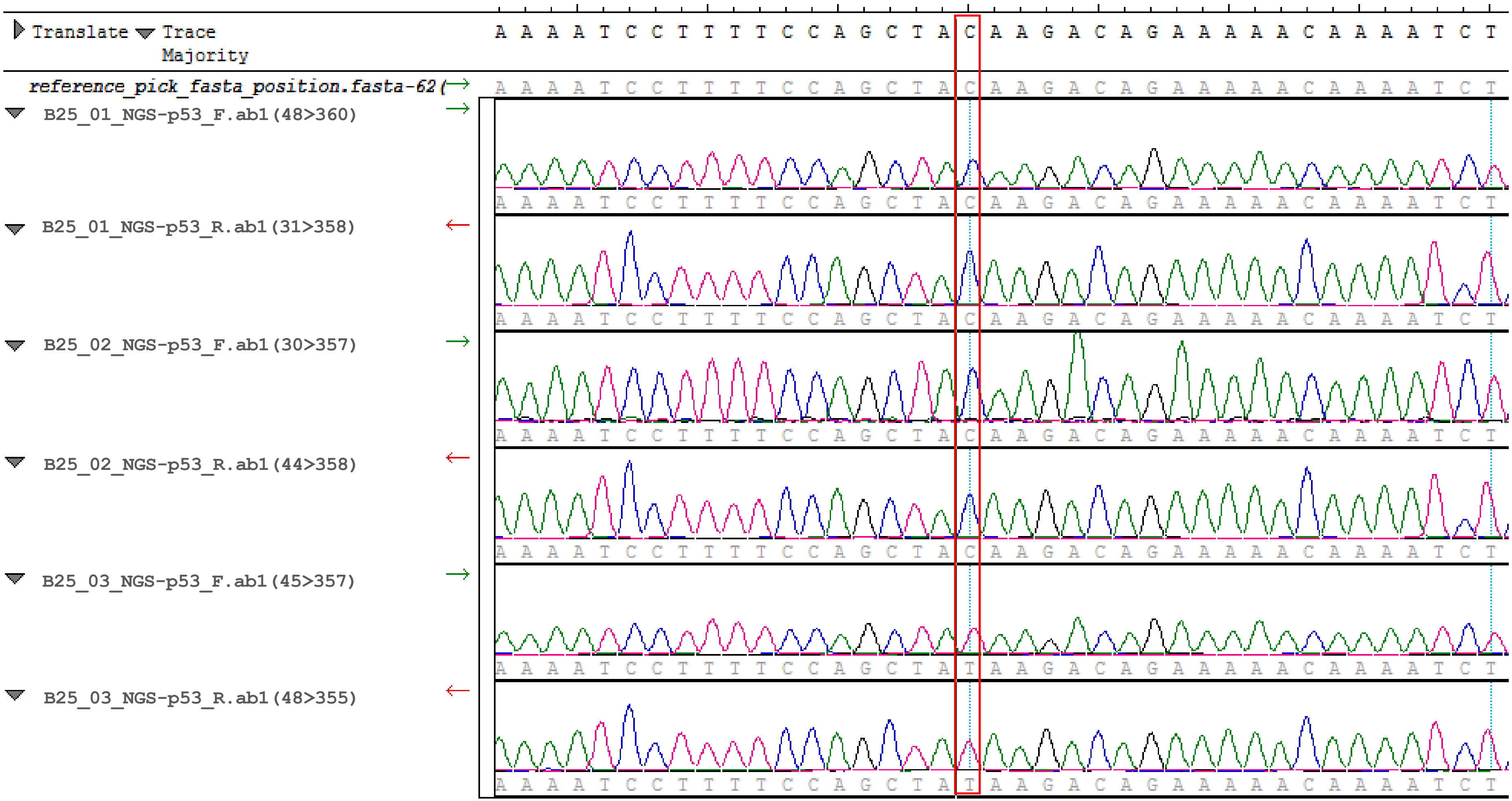

Translate
Trace
Majority
A A A A T C C T T T T C C A G C T A C A A G A C A G A A A A A C A A A A T C T
reference_pick_fasta_position.fasta-62(
B25_01_NGS-p53_F.ab1(48>360)
B25_01_NGS-p53_R.ab1(31>358)
B25_02_NGS-p53_F.ab1(30>357)
B25_02_NGS-p53_R.ab1(44>358)
B25_03_NGS-p53_F.ab1(45>357)
B25_03_NGS-p53_R.ab1(48>355)

GMIP

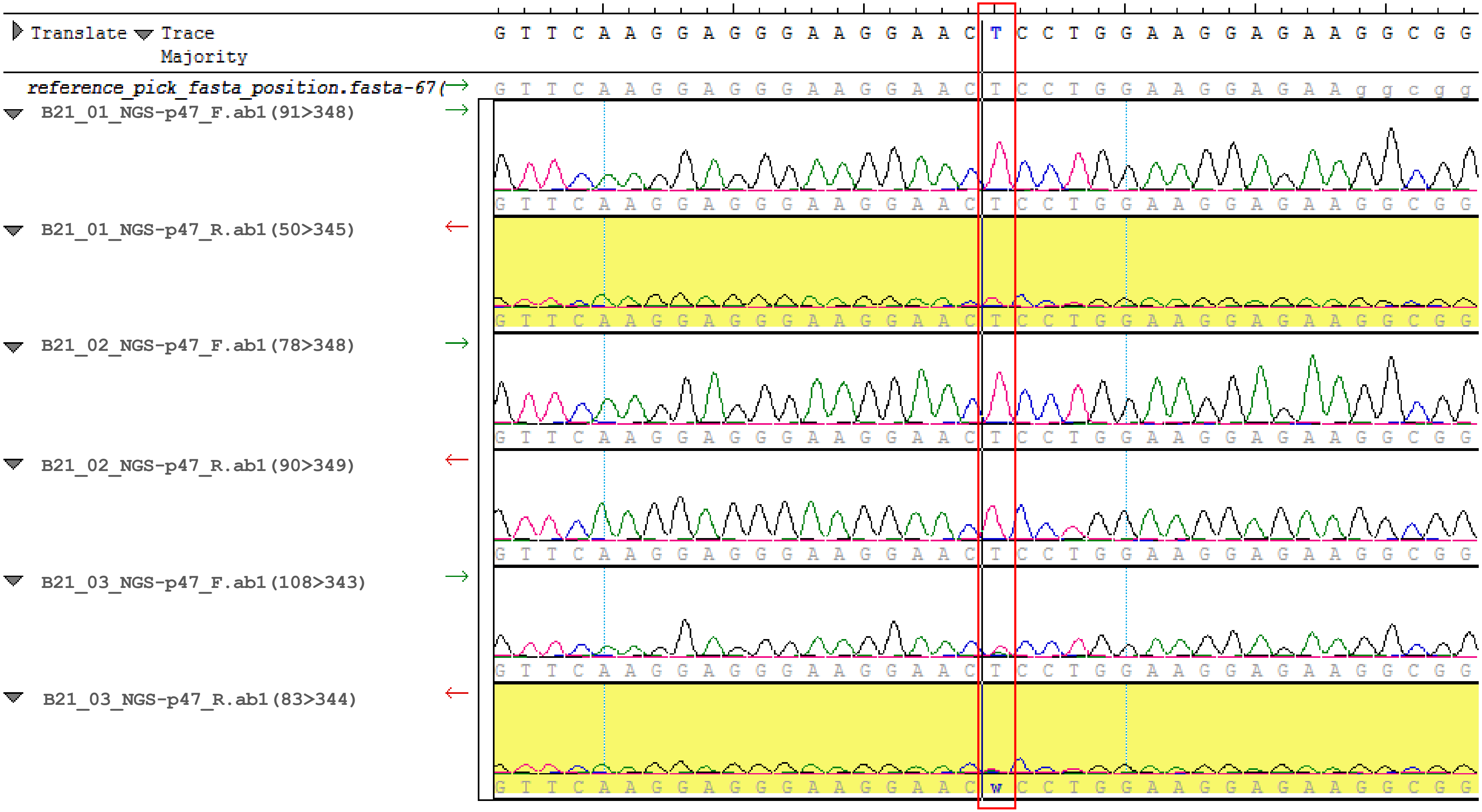
Translate
Trace
Majority
G T T C A A G G A G G G A A G G A A C T C C T G G A A G G A G A A G G C G G
reference_pick_fasta_position.fasta-67(
G T T C A A G G A G G G A A G G A A C T C C T G G A A G G A G A A g g c g g
B21_01_NGS-p47_F.ab1(91>348)
G T T C A A G G A G G G A A G G A A C T C C T G G A A G G A G A A G G C G G
B21_01_NGS-p47_R.ab1(50>345)
G T T C A A G G A G G G A A G G A A C T C C T G G A A G G A G A A G G C G G
B21_02_NGS-p47_F.ab1(78>348)
G T T C A A G G A G G G A A G G A A C T C C T G G A A G G A G A A G G C G G
B21_02_NGS-p47_R.ab1(90>349)
G T T C A A G G A G G G A A G G A A C T C C T G G A A G G A G A A G G C G G
B21_03_NGS-p47_F.ab1(108>343)
G T T C A A G G A G G G A A G G A A C T C C T G G A A G G A G A A G G C G G
B21_03_NGS-p47_R.ab1(83>344)
G T T C A A G G A G G G A A G G A A C W C C T G G A A G G A G A A G G C G G

ANKRD27

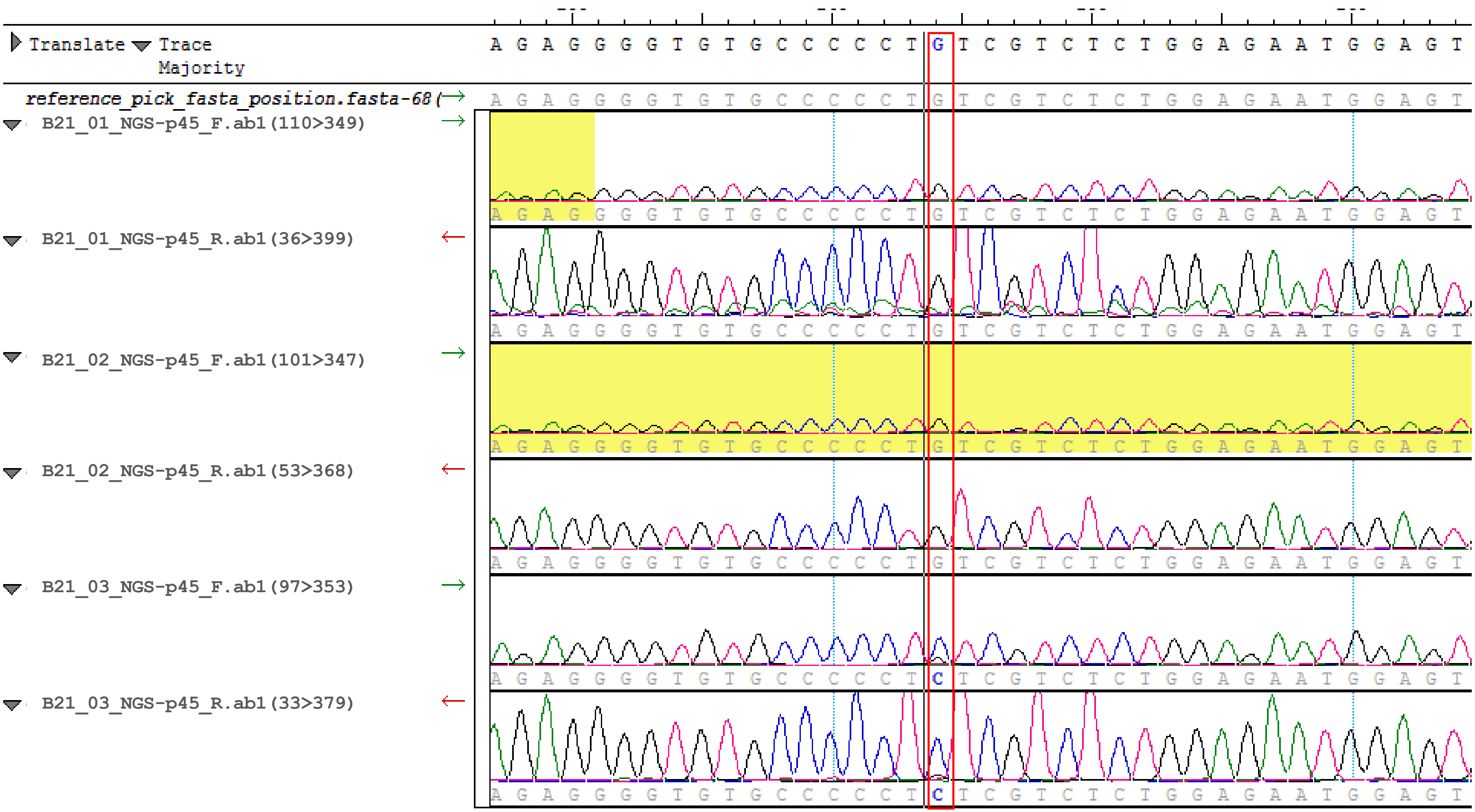

Translate
Trace
Majority
A G A G G G G T G T G C C C C C T G T C G T C T C T G G A G A A T G G A G T
reference_pick_fasta_position.fasta-68
B21_01_NGS-p45_F.ab1(110>349)
B21_01_NGS-p45_R.ab1(36>399)
B21_02_NGS-p45_F.ab1(101>347)
B21_02_NGS-p45_R.ab1(53>368)
B21_03_NGS-p45_F.ab1(97>353)
B21_03_NGS-p45_R.ab1(33>379)
A G A G G G G T G T G C C C C C T C T C G T C T C T G G A G A A T G G A G T

MYH14

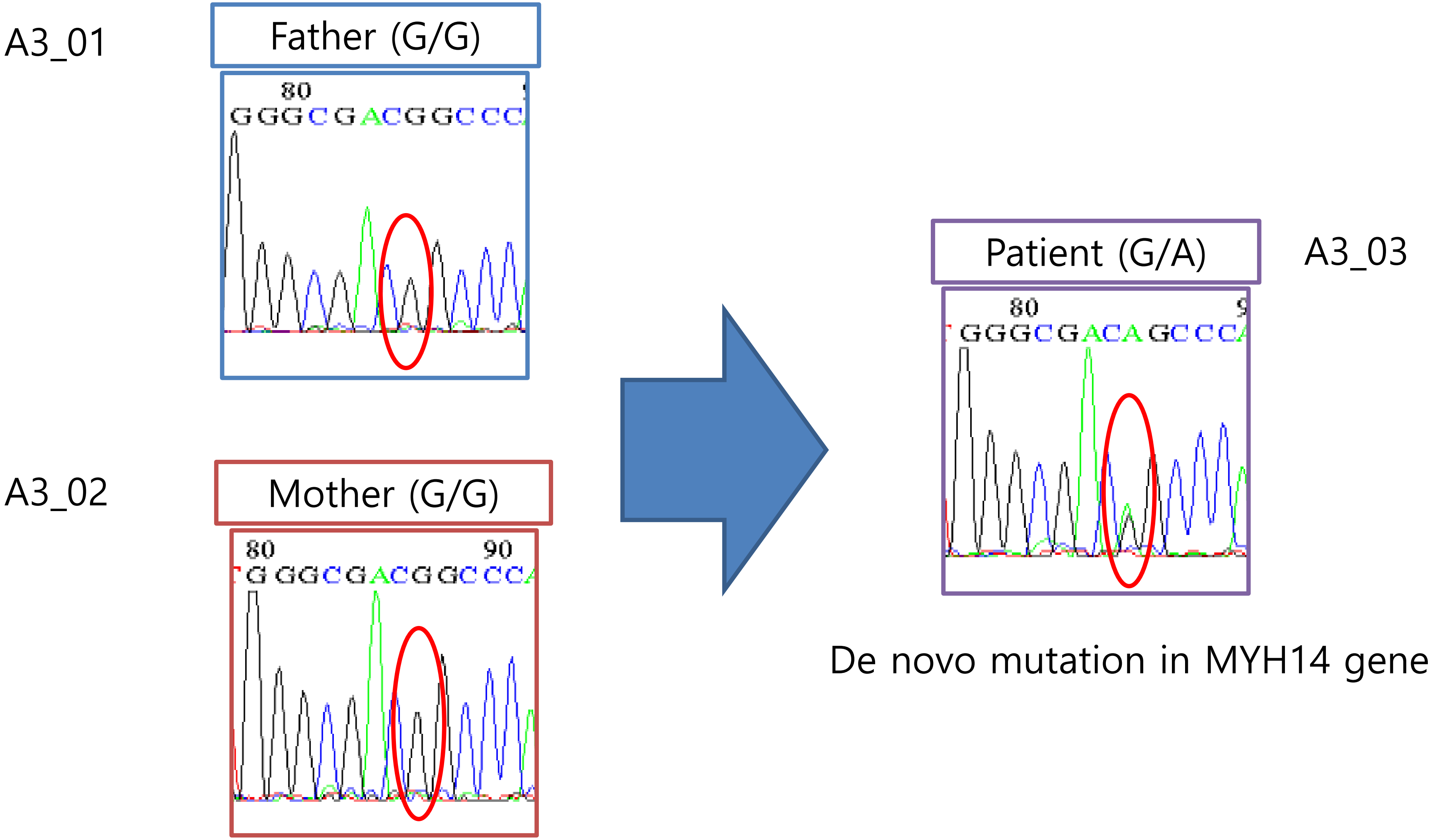

De novo mutation in MYH14 gene

TFPT

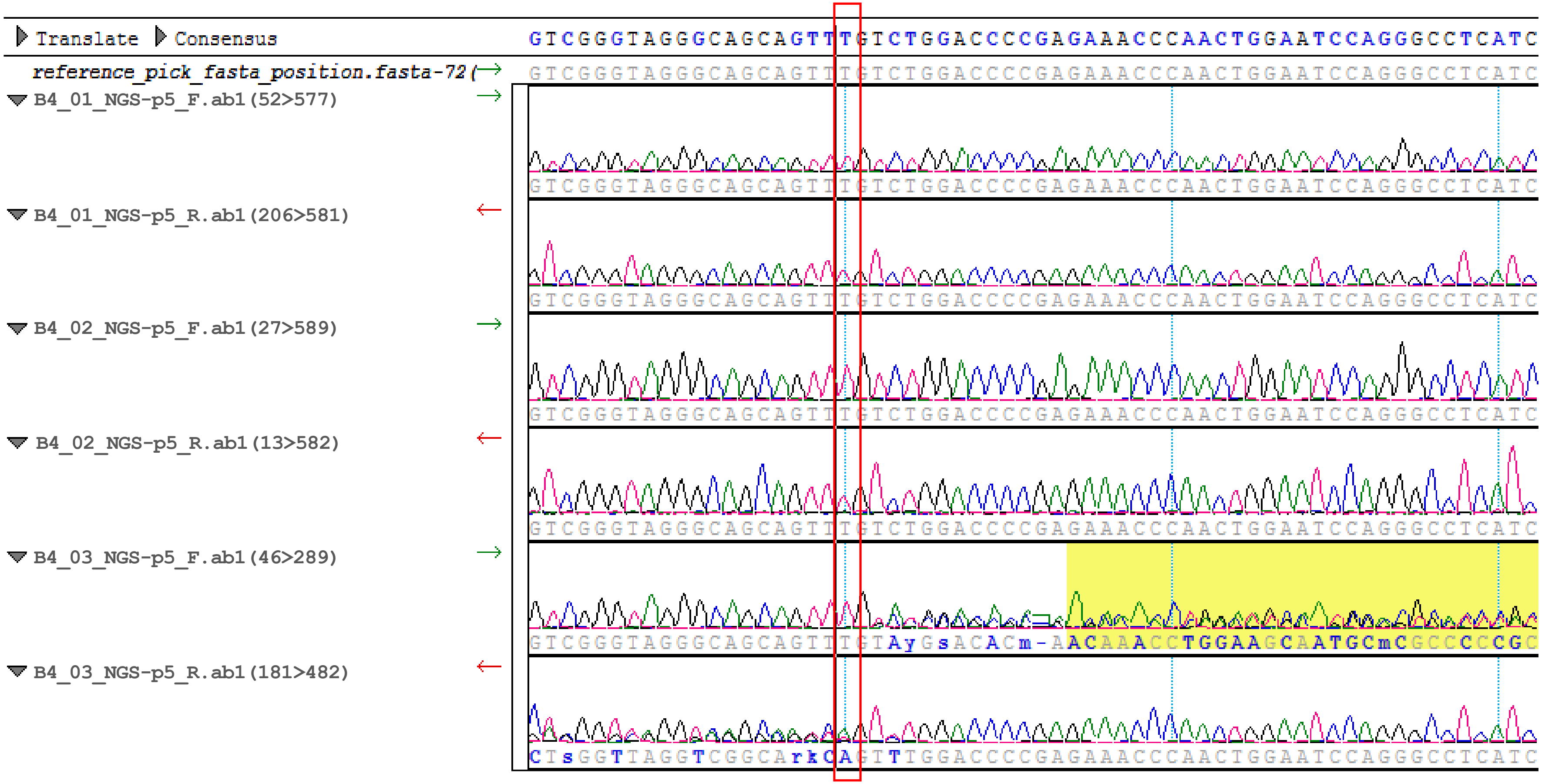

Translate
Consensus
GTCGGGTAGGGCAGCAGTTTGTCTGGACCCCGAGAAACCCAACTGGAATCCAGGGCCTCATC
reference_pick_fasta_position.fasta-72
B4_01_NGS-p5_F.ab1(52>577)
B4_01_NGS-p5_R.ab1(206>581)
B4_02_NGS-p5_F.ab1(27>589)
B4_02_NGS-p5_R.ab1(13>582)
B4_03_NGS-p5_F.ab1(46>289)
B4_03_NGS-p5_R.ab1(181>482)

NOL4L

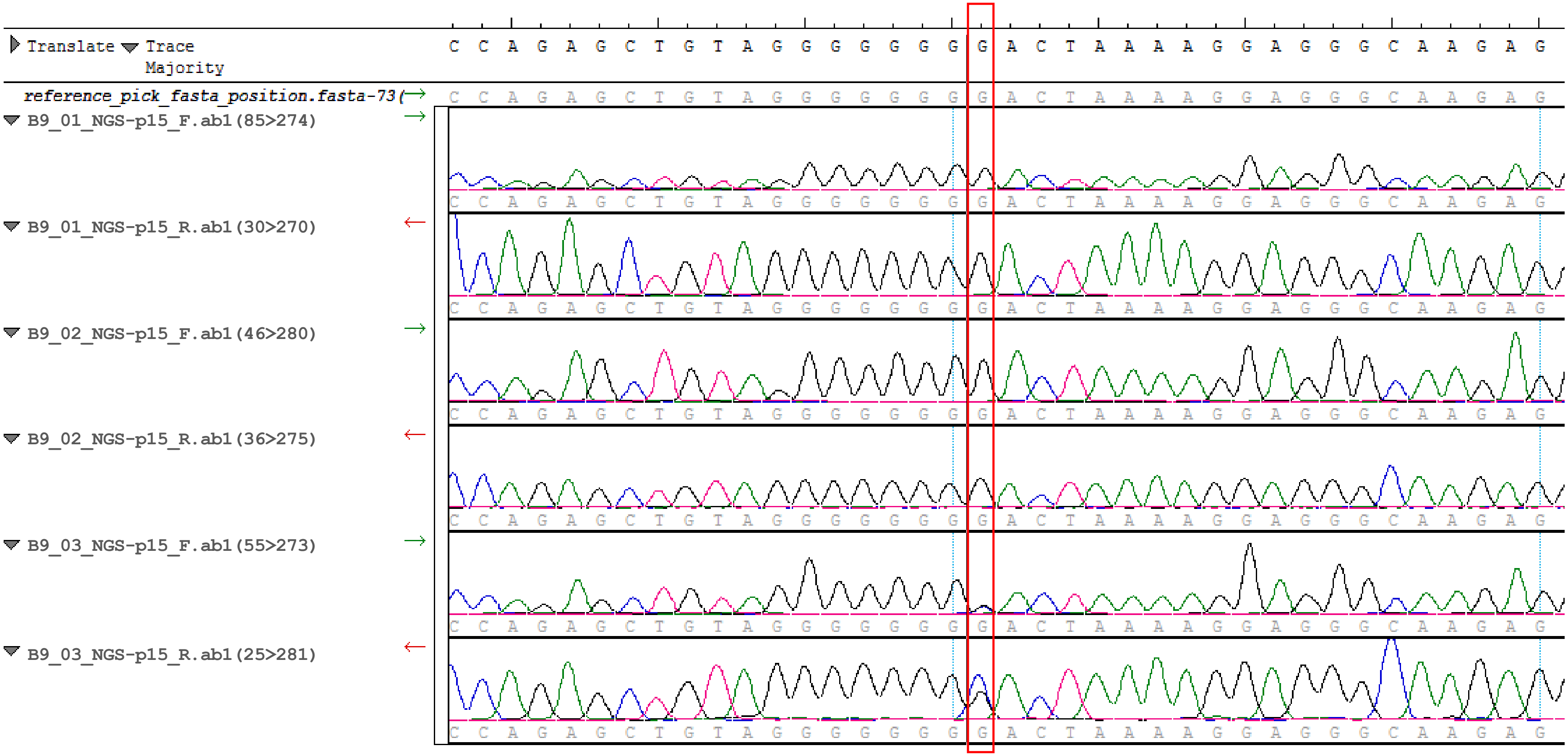

Translate
Trace
Majority
C C A G A G C T G T A G G G G G G G G A C T A A A A G G A G G G C A A G A G
reference_pick_fasta_position.fasta-73(
B9_01_NGS-p15_F.ab1(85>274)
B9_01_NGS-p15_R.ab1(30>270)
B9_02_NGS-p15_F.ab1(46>280)
B9_02_NGS-p15_R.ab1(36>275)
B9_03_NGS-p15_F.ab1(55>273)
B9_03_NGS-p15_R.ab1(25>281)

PPP1R16B

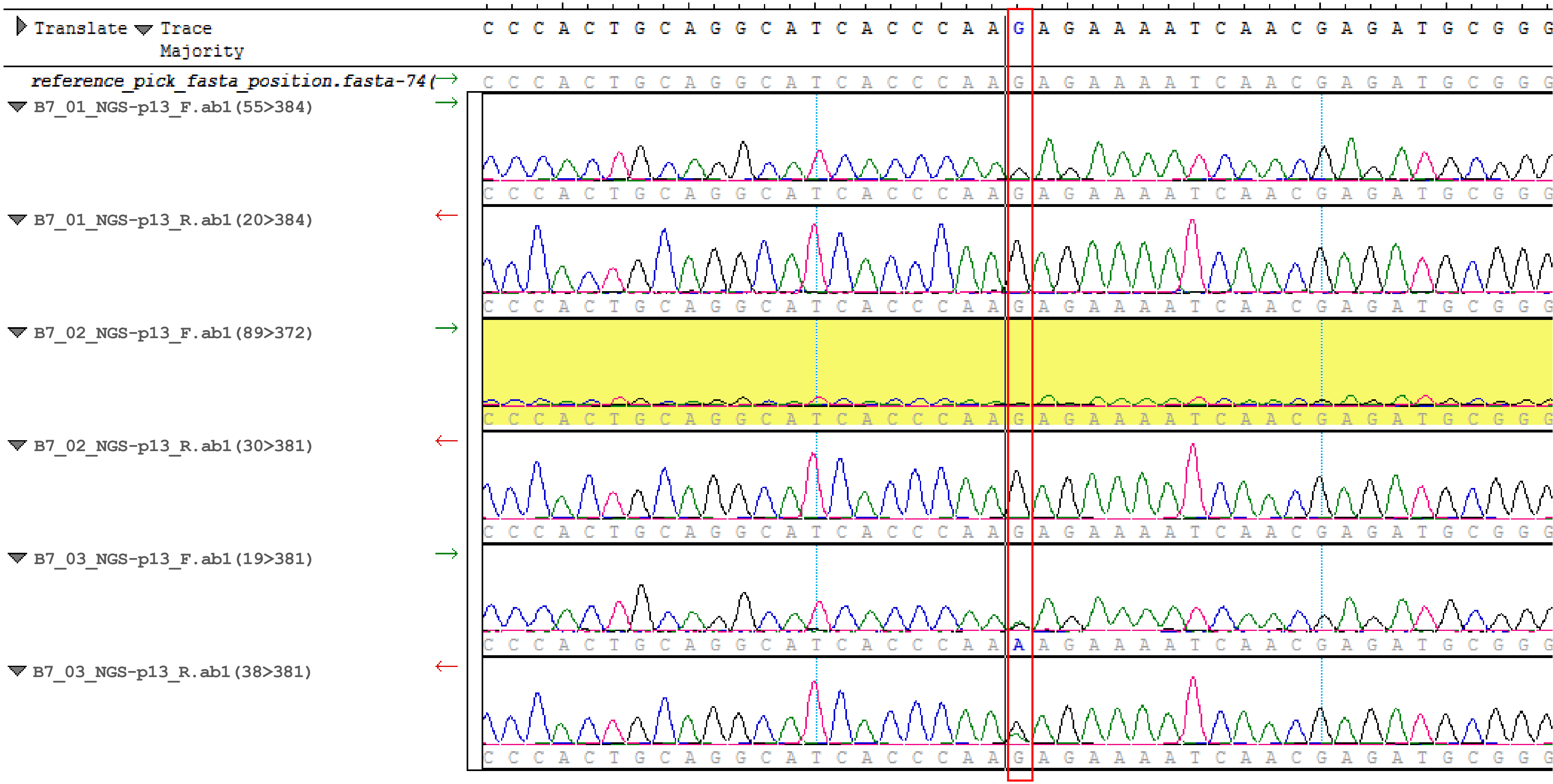

Translate
Trace
Majority
C C C A C T G C A G G C A T C A C C C A A G A G A A A A T C A A C G A G A T G C G G G
reference_pick_fasta_position.fasta-74(
B7_01_NGS-p13_F.ab1(55>384)
B7_01_NGS-p13_R.ab1(20>384)
B7_02_NGS-p13_F.ab1(89>372)
B7_02_NGS-p13_R.ab1(30>381)
B7_03_NGS-p13_F.ab1(19>381)
B7_03_NGS-p13_R.ab1(38>381)

COL6A2

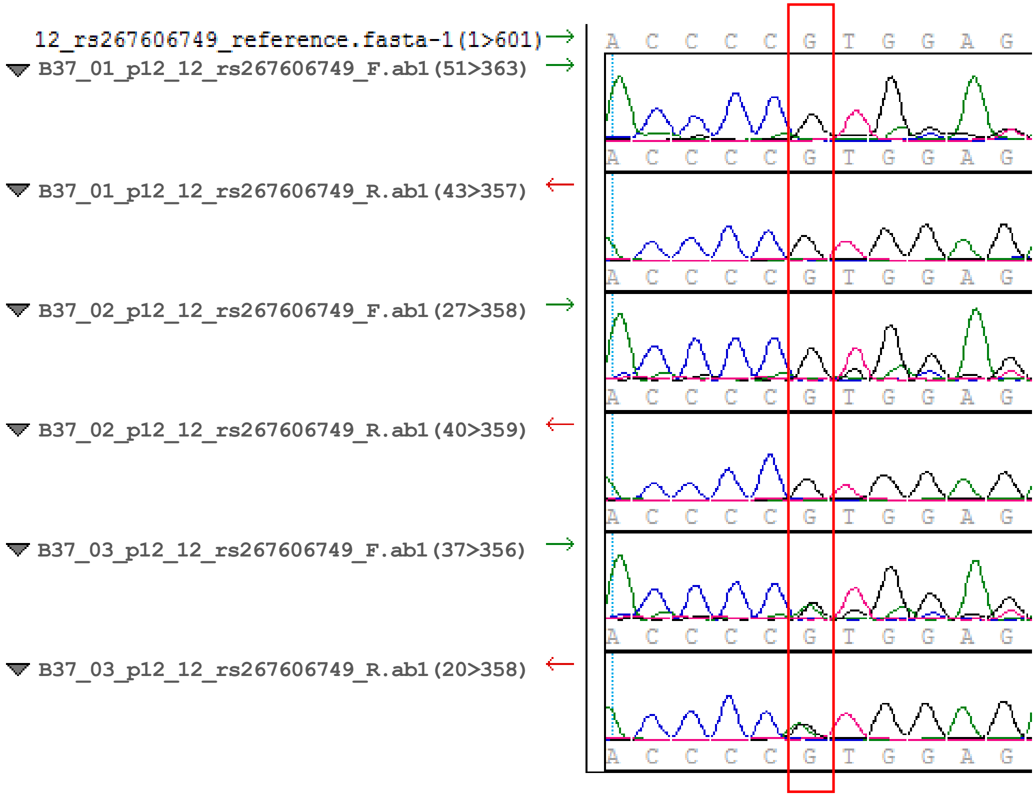
12_rs267606749_reference.fasta-1(1>601)
B37_01_p12_12_rs267606749_F.ab1(51>363)
B37_01_p12_12_rs267606749_R.ab1(43>357)
B37_02_p12_12_rs267606749_F.ab1(27>358)
B37_02_p12_12_rs267606749_R.ab1(40>359)
B37_03_p12_12_rs267606749_F.ab1(37>356)
B37_03_p12_12_rs267606749_R.ab1(20>358)
A C C C C G T G G A G

HMGXB4

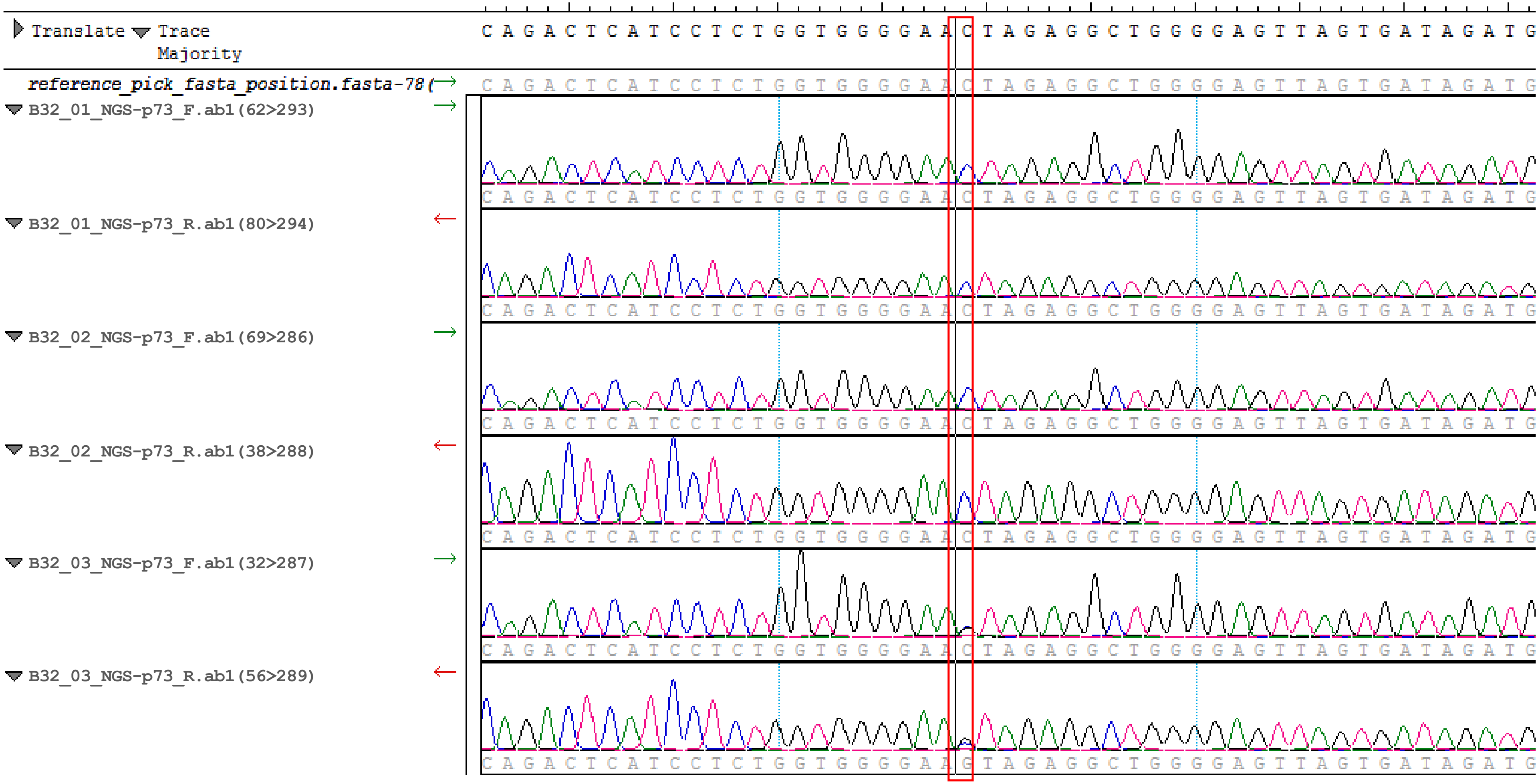

Translate
Trace
Majority
C A G A C T C A T C C T C T G G T G G G G A A C T A G A G G C T G G G G A G T T A G T G A T A G A T G
reference_pick_fasta_position.fasta-78 (
B32_01_NGS-p73_F.ab1(62>293)
B32_01_NGS-p73_R.ab1(80>294)
B32_02_NGS-p73_F.ab1(69>286)
B32_02_NGS-p73_R.ab1(38>288)
B32_03_NGS-p73_F.ab1(32>287)
B32_03_NGS-p73_R.ab1(56>289)

MT-ND2

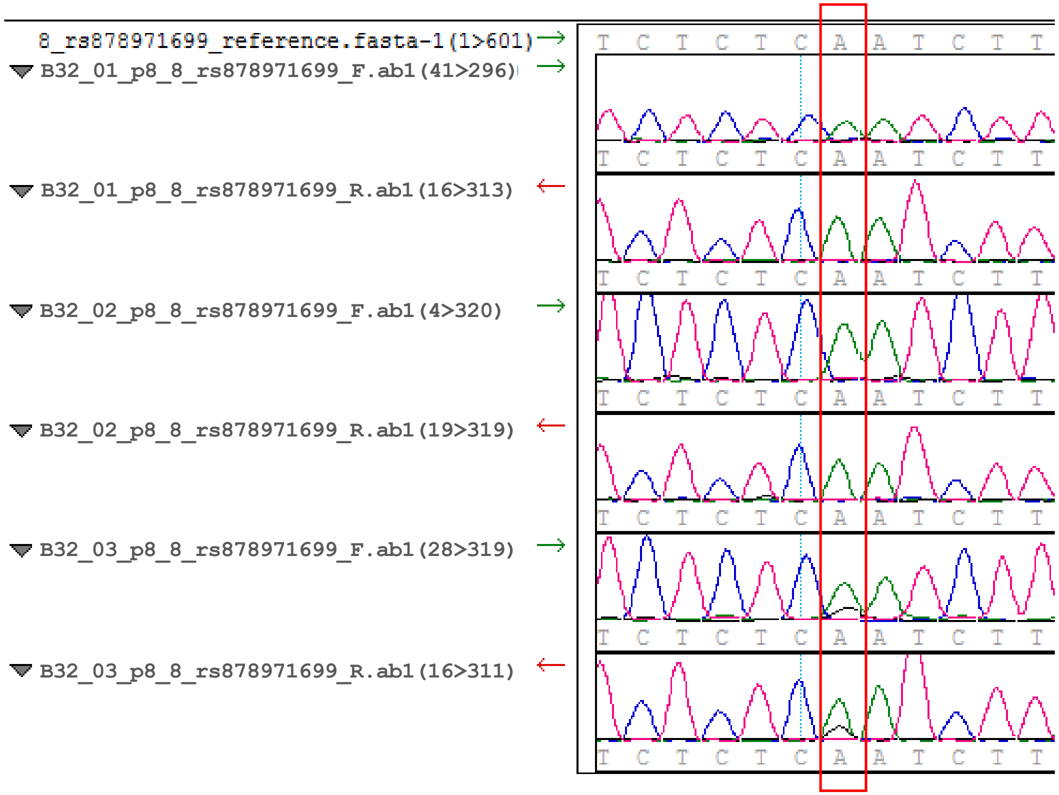
8_rs878971699_reference.fasta-1(1>601)
B32_01_p8_8_rs878971699_F.ab1(41>296)
B32_01_p8_8_rs878971699_R.ab1(16>313)
B32_02_p8_8_rs878971699_F.ab1(4>320)
B32_02_p8_8_rs878971699_R.ab1(19>319)
B32_03_p8_8_rs878971699_F.ab1(28>319)
B32_03_p8_8_rs878971699_R.ab1(16>311)
T C T C T C A A T C T T
